# Basin-scale multi-decadal analysis of hydraulic fracturing and seismicity in western Canada shows non-recurrence of induced runaway fault rupture

**DOI:** 10.1038/s41598-022-18505-0

**Published:** 2022-08-24

**Authors:** Germán Rodríguez-Pradilla, David W. Eaton, James P. Verdon

**Affiliations:** 1 Department of Geoscience, University of Calgary, Calgary, AB Canada; 2 Present Address: School of Earth Sciences, University of Bristol, Bristol, UK

**Keywords:** Seismology, Natural gas

## Abstract

Hydraulic fracturing (HF) is a reservoir stimulation technique that has been widely deployed in recent years to increase the productivity of light oil and/or natural gas from organic-rich, low-permeability formations. Although the process of fracturing a rock typically results in microseismic events of magnitude < 0, many cases of felt seismic events (typically magnitude 3.0 or larger) have also been reported. In the Western Canada Sedimentary Basin (WCSB), where more than 40,000 wells have been drilled and hydraulically fractured in the past two decades, the occurrence of HF-induced events has surged in some areas. Yet, many other areas of the WCSB have not experienced a significant increase in induced seismicity, despite a sharp increase in both the number of HF wells and the volumes of injected fluid. The relationship between injected volume and induced magnitudes can be quantified using the seismic efficiency ratio (S_EFF_), which describes the ratio between the net seismic moment release and the injected fluid volume. Runaway rupture, in which the fault rupture is dominated by the release of accumulated tectonic stresses, is inferred to be marked by an abrupt increase in S_EFF_ to a value > 0.5. Most previous studies of induced earthquakes have been limited to a single operation and/or seismicity sequence. To better understand the observed variability of the seismic response to HF stimulations at a basin scale, we compiled HF data for all unconventional wells hydraulic fractured in the WCSB between 2000 and 2020, together with the seismicity reported during the same period. We grouped these observations into bins measuring 0.2° in longitude and 0.1° in latitude, or approximately 13 by 11 km. We identified 14 areas where large magnitude events resulted in high S_EFF_ values, implying runaway rupture had taken place. However, we find that in these areas, sustained fluid injection did not lead to persistent high S_EFF_ values. Instead, as injection continued, S_EFF_ values returned to values less than 0.5. This suggests that there is a limited budget of tectonic strain energy available to generate runaway rupture events: once this is released, event magnitudes decrease even if high volume injection persists.

## Introduction

Hydraulic fracturing (HF) is a reservoir stimulation technique that has been extensively used to enhance the production of hydrocarbons from organic-rich, low-permeability shale formations. A causal association between hydraulic fracturing and induced (anthropogenic) earthquakes has been documented around the world^1^, including in the Bowland Shale in the UK^2,3^, the Sichuan Basin in China^4^, the Utica Shale in Ohio^5^, the Woodford Shale in Oklahoma^6^, and the Montney^7^ and Duvernay^8^ Formations in the Western Canada Sedimentary Basin (WCSB).

**Figure 1:**
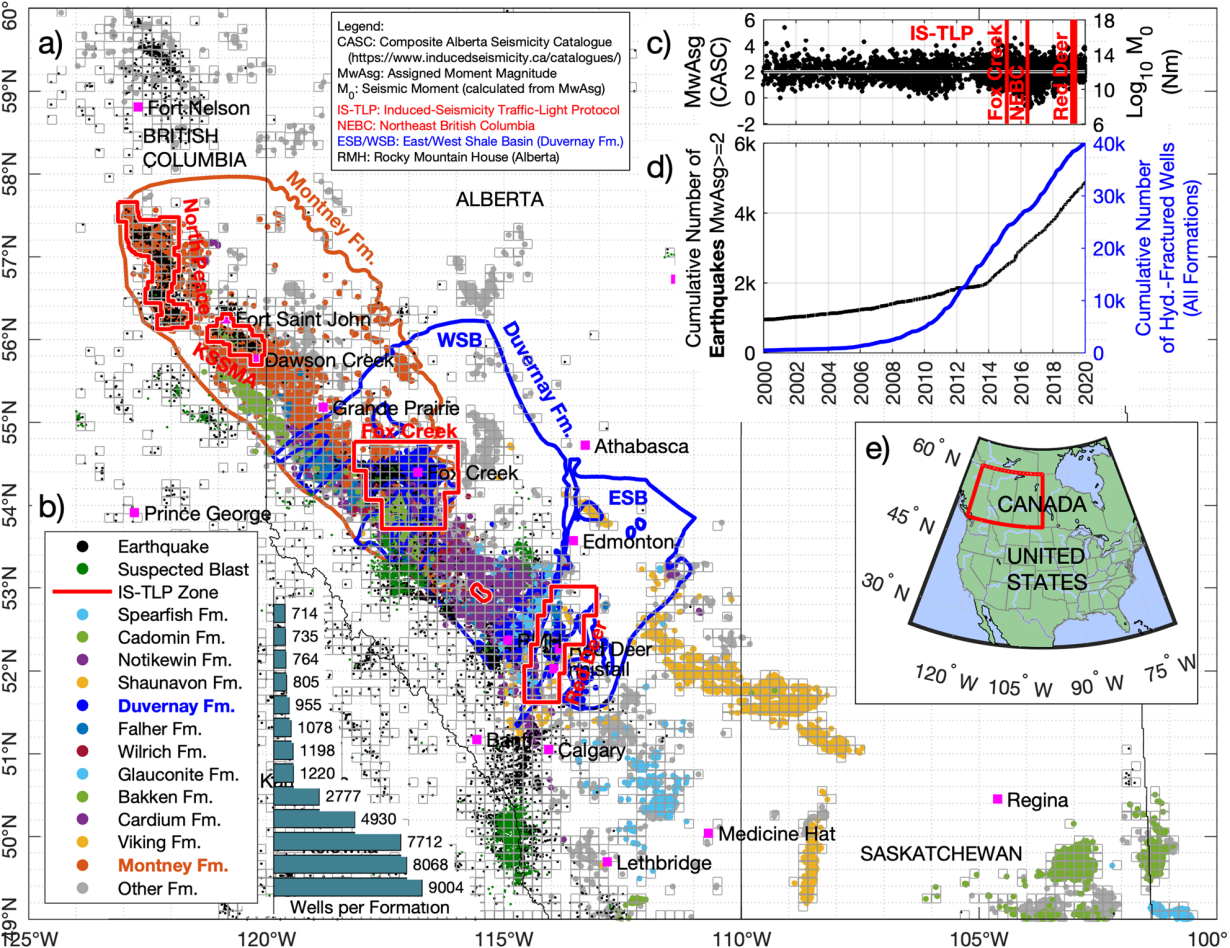
Hydraulically fractured (HF) wells in the Western Canada Sedimentary Basin (WCSB) between January 1, 2000 and January 1, 2020, and the reported seismicity during the same period. The HF wells, colour-coded by the stimulated formation, together with the boundary of the Duvernay and Montney formations, are shown in (**a**), and the number of stimulated wells per formation is shown in (**b**). The moment magnitude of earthquakes and suspected blasts from the compiled public seismic catalogs^45,49^ are shown in (**c**), together with the date of the implementation of magnitude-based traffic-light protocols for induced seismicity (IS-TLP) in North Peace and the Kiskatinaw Seismic Monitoring and Mitigation Area (KSMMA) areas for the Montney Formation, and Fox Creek and Red Deer areas for the Duvernay formation (red vertical lines). The strong correlation between the number of hydraulic fractured wells and the number of seismic events, shown in (**d**), is the main motivation of this study. (**e**) Reference map of North America, with the studied area shown in (**a**) highlighted in red. The number of suspected blasts has also increase in western Canada since 2014; these were removed from the seismicity catalog prior to calculating response paths. The HF wells and earthquakes shown in (**a**) were grouped into bins measuring 0.2° in longitude and 0.1° in latitude (approximately 11 × 13 km) to calculate the total fluid pumped, total seismic moment and maximum earthquake magnitude inside each bin, as shown in Fig. 2. The temporal variation of the seismic and hydraulic fracturing activity illustrated in this figure is also shown in Video S1 in the supplementary materials. All geoLOGIC systems ltd. data and software is copyright 2022.

**Figure 2:**
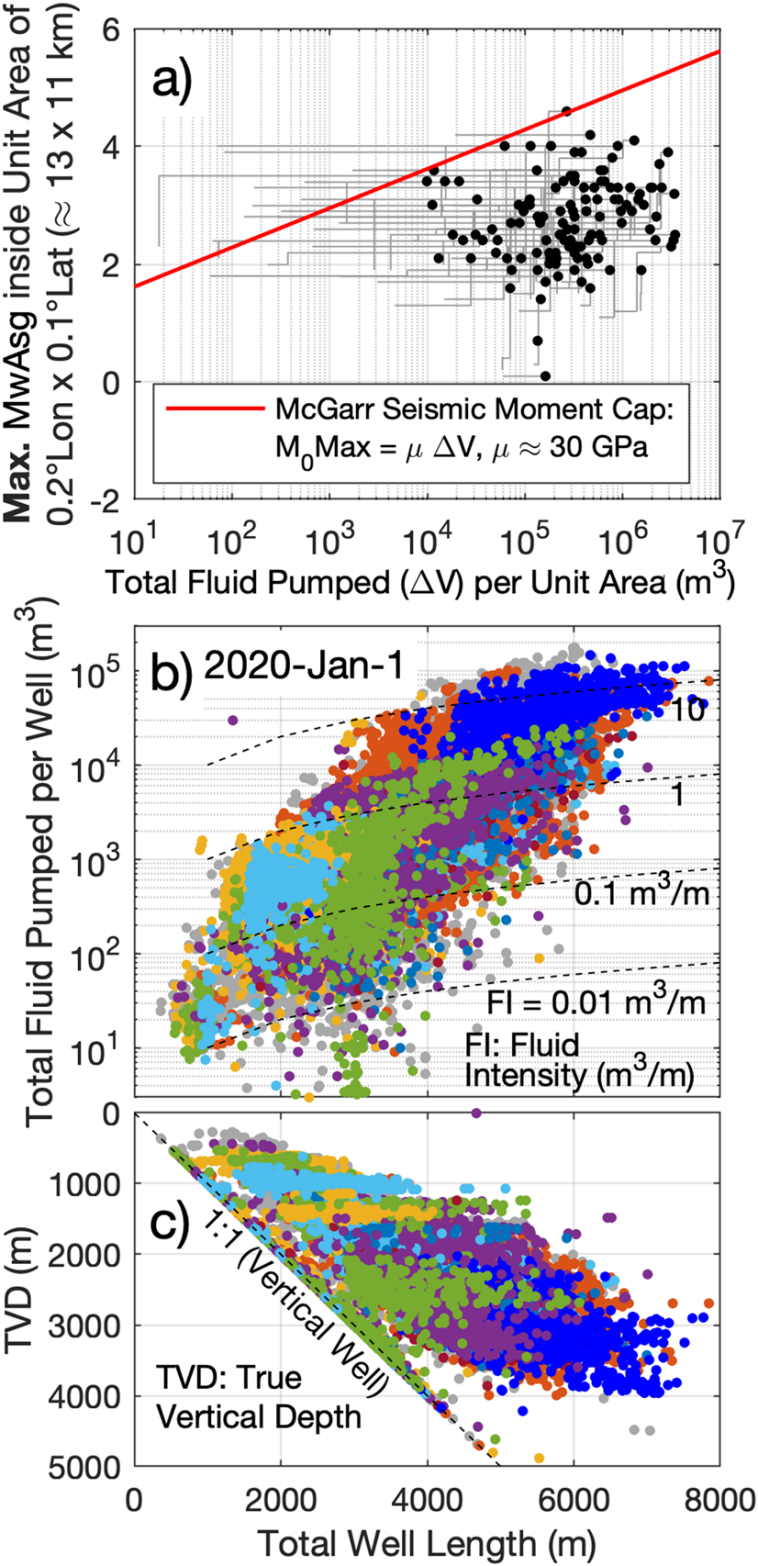
(**a**) Total fluid pumped (TFP) in every unit area of 0.2° longitude by 0.1° latitude (the unit areas are shown in Fig. 1a), and the moment magnitude of the seismic events (shown in Fig. 1c). The black dots show the maximum magnitude and total volume as of January 1, 2020 reported inside each unit area that contains hydraulically fractured (HF) wells, for seismic events that occurred after the start of HF operations and up to 90 days after completion. The grey lines show the response path, which tracks the magnitude-volume relationship over time. Although almost every black dot falls approximately at or below the McGarr moment cap^25^ marked by the red line, 14 response paths significantly surpass this relationship. In 93% of such cases (13 out of the 14 cases), continued fluid injection within the same unit area as the induced earthquake did not trigger another large seismic event. (**b**) Total well length (TWL) or measured depth (MD) of the HF wells, with the true vertical depth and total fluid pumped (TFP) during the HF stimulation of each well until January 1st, 2020 (**c**). The fluid intensity of the hydraulic stimulation (i.e., TFP/TWL, in m^3^/m), is also shown in (**b**). The temporal variation of the total fluid pumped per HF well and maximum moment magnitudes illustrated in this figure is also shown in Video S1 in the supplementary materials. All geoLOGIC systems ltd. data and software is copyright 2022.

In the Western Canada Sedimentary Basin (WCSB), more than 40,000 wells have been drilled and hydraulically fractured between the years 2000 and 2020 (Fig. 1)^9^. Over this time, there has been a progressive increase in well depth, as deeper formations are explored, and an increase in the length of the lateral sections of horizontal wells. Taken together, this has led to higher volumes of fluid pumped into each HF well (Fig. 2). Many areas of the WCSB have not experienced any significant increase in seismicity, despite a large increase in both the number of hydraulically fractured wells and the volumes of fluid pumped^10^. However, in certain areas of western Canada, particularly near Fox Creek^11^ and Red Deer^12^ in central Alberta, and in the Horn River Basin^13^ and Fort St. John area in NE British Columbia^14^, the rate of seismicity has grown in conjunction with increasing intensity of hydraulic-fracturing operations. This spatial correlation has prompted provincial agencies to introduce new regulations, most notably Traffic Light Protocols (TLPs) to manage the risk of induced seismicity^15,16^.

Given the impacts of induced seismicity, both on hydrocarbon producers and on the public who live near to their operations^17^, much recent research has focussed on the factors that control the magnitudes of induced earthquakes^2,18,19^. Broadly speaking, two end-member scenarios have been proposed. In the arrested rupture scenario, events primarily release strain that has been introduced by the industrial activity, and hence the maximum magnitude is limited by the injection volume^20–22^. In the runaway rupture scenario, the fault rupture is initiated by the industrial activity, but it extends beyond the fluid-perturbed region and releases tectonically accumulated strain energy. In this scenario, the maximum magnitude is limited only by tectonic factors^23^.

In 1976, McGarr^24^ developed a formulation to relate the volume of rock and/or fluid extracted during mining to the maximum seismic moment released as induced seismicity. In 2014, McGarr^25^ adapted this formulation to estimate the maximum seismic moment release generated by subsurface fluid injection. The McGarr equation, and the resulting upper bound for seismic moment release, is based on several assumptions, including that all of the strain released by the seismicity is directly generated by the subsurface deformation induced by industrial activities. However, detailed microseismic observations of induced seismicity sequences indicate that hydraulic fracturing induced seismicity occurs on pre-existing tectonic faults, and therefore may be releasing stored tectonic strain energy, in addition to any strain imparted by the injection operations^26,27^. Atkinson et al.^10^ presented a selection of case studies from the WCSB where induced earthquake magnitudes appear to exceed the McGarr maximum magnitude based on volumes in HF wells, indicating that runaway rupture had occurred, whereas the induced earthquakes from water disposal wells in the same basin did not exceed McGarr’s maximum magnitude cap. Similarly, seismicity at the Pohang enhanced geothermal site in South Korea appears to have significantly exceeded the McGarr volume-based maximum magnitude^28^.

In this “runaway rupture” scenario^28^, the injection creates a perturbation that initialises earthquake rupture within the stimulated rock volume, but this rupture extends along larger faults into rock volumes that are unaffected by injection-induced pressure changes. In this scenario, the upper limit to induced earthquake magnitudes is controlled by the structural, geomechanical and tectonic characteristics of the formation in question, rather than by the scale of the injection operations. We note that even in a runaway rupture scenario, assuming a rate and state nucleation model, earthquake rate is still expected to scale with stressing rate, which in turn might be expected to scale with injection volume. Therefore, a scaling or correlation between injection volume and seismicity should still be expected in a runaway rupture scenario, but with no upper bound to the value of any scaling coefficient. We also note in passing that this scenario does not necessarily imply that the maximum magnitude, M_MAX_, for induced seismicity will be the same as tectonic M_MAX_ estimates, since the formations targeted for hydraulic fracturing typically have lower stresses, and smaller faults, than mid-crustal rocks where larger earthquakes are typically generated^29^, which are the primary control on tectonic M_MAX_ estimates.

The phenomenon of runaway rupture may impose its own limits on the amount of seismic moment released over the longer term. Tectonic strain energy is accumulated over geological timescales, meaning that there is no opportunity for this energy to be recharged over the timescales in which hydraulic fracturing takes place (years and/or decades). In principle, once this energy has been released, further injection in the vicinity of a reactivated fault will only be able to release the strain directly imparted by injection. Given the above, we might expect to observe the following with respect to earthquake magnitudes. During initial operations, tectonic strain energy can be released, leading (in some areas and some formations, where critically-stressed faults are present) to rapid escalation of event magnitudes. However, as injection continues within an area and the tectonic strain energy budget has been consumed, the cumulative seismic moment would be expected to revert to the bounds imposed by a volumetric cap. Alternatively, if the accumulated tectonic strain budget is sufficiently high, runaway rupture may persist for extended periods of time.

In this study, we examine and compare the temporal evolution of induced earthquake magnitudes and injection volumes in hydraulic fracturing wells in the WCSB over the past two decades. The availability of hydraulic fracturing and induced seismicity data from the WCSB, a region which covers over 1000 km from north to south, provides an opportunity to evaluate the hypotheses described above at a regional (basin) scale.

## Runaway rupture and volume-based magnitude limits

McGarr^24^ proposed a relationship between the cumulative seismic moment (ΣM_0_) of induced seismic events observed in oilfields and copper mines in the 1960s and 1970s, based on the volume changes (|ΔV|) created either by injection or extraction of fluids, or removal of rock volumes by mining. McGarr^25^ refined this relationship for cases of seismicity induced by the injection of large volumes of fluid—mostly saltwater disposal, enhanced geothermal systems (EGS) and unconventional hydrocarbon wells. In addition to the relationship between ΔV and ΣM_0_

1 $$\sum {M}_{0}=2\mu \left|\Delta V\right|$$

McGarr^25^ further derived the expected moment of the largest induced event, M_0_(max), by assuming an earthquake stress drop equal to half of the stress buildup during a tectonic cycle and a *b*-value of 1 for the Gutenberg-Righter distribution^30^ of the magnitudes of the induced events:

2 $${M}_{0}\left(\mathrm{max}\right)=\mu \Delta V$$

The maximum moment magnitude of induced events, M_MAX_, can be calculated with the moment-magnitude scale from Hanks and Kanamori ${M}_{MAX}=({\mathrm{log}}_{10}{M}_{0}\left(\mathrm{max}\right)-9.05)/1.5$^31^.

In addition to the above-referenced assumptions, the McGarr relationship represents an upper bound, in that it assumes that the strain generated by subsurface injection is released as seismic energy. In reality, for many sites, much of the deformation may be released as aseismic deformation (for example, by creep on fractures and/or poroelastic expansion of the reservoir rocks). As a result, for most injection sites, ΣM_0_ falls well below the McGarr limit, and ${M}_{0}\left(\mathrm{max}\right)\ll \mu \Delta V$. To account for this, Hallo et al.^21^ proposed a modification to the McGarr relationship, introducing a Seismic Efficiency Ratio (S_EFF_) parameter as a calibration factor to McGarr’s relationship,

3 $$\sum {M}_{0}={S}_{EFF} \mu \left|\Delta V\right|$$

S_EFF_ has been observed to range between 10^–6^, to a ratio of unity (or higher)^21^. Noting that Eq. (2) assumes a stress drop corresponding to 50% of the maximum stress drop over a seismic cycle, Li et al.^32^ derived a physical interpretation for S_EFF_,

4 $${S}_{EFF} = \frac{1}{2(1-c)}$$

where *c* is the fraction of the full co-seismic stress drop during a tectonic loading cycle. This formulation implies that S_EFF_ ≥ 0.5 and has no upper limit. A value of S_EFF_ that is less than 0.5 does not require the release of any tectonic strain energy; rather, it indicates the prevalence of deformation processes associated with fluid injection^21^. Hence, hereafter, we refer to the situation whereby induced earthquake magnitudes are limited by the injected volume, as per McGarr^25^, as the arrested-rupture scenario, since the implication is that rupture dimensions, and hence earthquake magnitudes, are limited by the injection volume. We note that, in practice, a sequence of induced seismicity may release both tectonically stored energy and the injection-induced strain at a rate that produces an overall S_EFF_ < 0.5. In such situations, discriminating between scenarios will be difficult in practice, unless high-resolution microseismicity observations are available from which deformation processes can be imaged in detail.

Galis et al.^22^ proposed an alternative model for arrested rupture cases where

5 $${M}_{0}\left(max\right)=\gamma\Delta {V}^{3/2},$$

in which γ is determined by the reservoir thickness, bulk modulus and coefficient of dynamic friction. However, the Galis et al. model is based on an assumption that the injection-induced perturbation can be represented as an expanding cylinder within the reservoir^22^, a situation that is unlikely to be representative of hydraulic fracturing in shale reservoirs, where the evolution and distribution of pressures may be strongly controlled by, for example, the presence of permeable structures such as fracture networks within the reservoir^27^.

The alternative endmember to the arrested rupture scenario is that induced earthquakes, while initiated by injection, generate ruptures that release significant quantities of stored tectonic strain energy. We refer to this as the runaway rupture scenario. Within the Hallo et al. S_EFF_ framework, a runaway rupture scenario would be represented by values of S_EFF_ that exceed 0.5, implying that the seismic moment released exceeds the amount of deformation created by the injection, and therefore that a significant portion of the seismic moment is generated by the release of tectonic strain energy. The behaviour of such cases over an extended period of injection then becomes of particular significance. If, for a given area, we initially observe runaway rupture and S_EFF_ > 0.5, but over time S_EFF_ trends back to 0.5 or lower, then we can assume that initial seismicity was dominated by the release of tectonic energy (i.e., runaway rupture), but that the tectonic strain energy budget was limited, and that subsequent injection is dominated by arrested ruptures, and is not able to generate seismicity at a rate that exceeds the McGarr cap.

**Figure 3:**
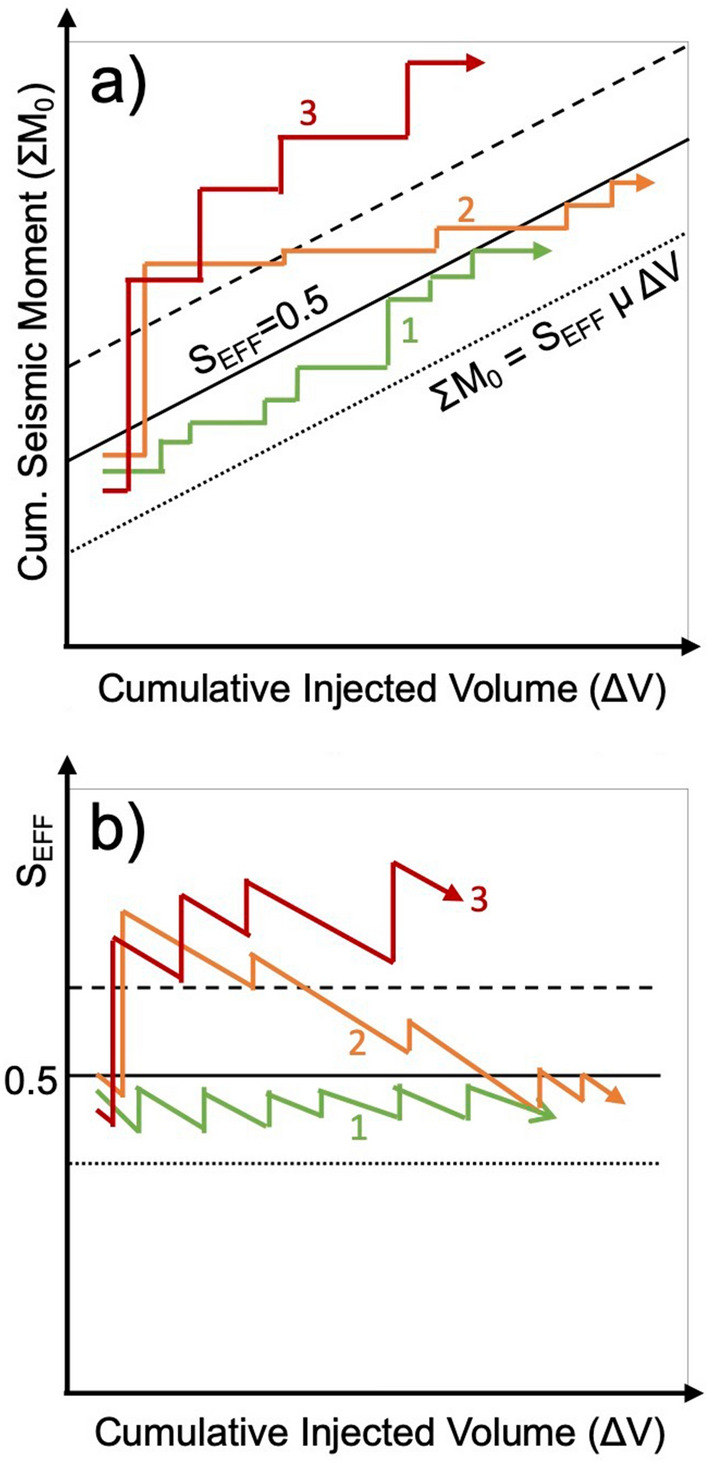
Schematic illustrations of conceptual induced seismicity response paths. In (**a**) we show the potential evolution of ΣM_0_ relative to ΔV for three conceptual scenarios. Scenario 1 (in green) shows an arrested-rupture case, where magnitudes are always limited by the injection volume according to Eq. (3), with S_EFF_ = 0.5. Scenario 2 (orange) shows a runaway rupture case, where ΣM_0_ exceeds this cap given by Eq. (3). However, over time, once the tectonic strain energy budget has been released the cumulative seismic moment relationship reverts to that described by Eq. 3. Scenario 3 (red) shows a runaway rupture case where the tectonic strain energy continues to contribute to the induced seismicity over an extended period, with ΣM_0_ continuing to exceed the Eq. (3) cap. In (**b**) we show the same conceptual scenario paths viewed with respect to S_EFF_.

Figure 3 shows schematic examples of conceptual response paths of seismic activity according to the scenarios described in the preceding paragraphs. Scenario 1 shows a situation with a relatively stable S_EFF_ around 0.5, at the upper limit for propagation of arrested ruptures^22^, following the relationship described by Eqs. (3 and 4). Scenarios 2 and 3 show sharp increases in the seismic activity during injection operations that exceed the McGarr cap, implying the occurrence of runaway rupture and the release of significant amounts of stored tectonic strain energy. In Scenario 2, the contribution from the tectonic strain energy is then used up, and the evolution of magnitudes returns to that described by the McGarr cap. In Scenario 3, ΣM_0_ continues to exceed the McGarr cap over an extended period of time, implying a continued contribution to the overall seismic strain release from tectonic strain. Figure 3b shows the evolution of these scenarios with respect to S_EFF_: in the arrested rupture case (Scenario 1), S_EFF_ is below 0.5 throughout; in Scenario 2, S_EFF_ is initially greater than 0.5, but then drops below 0.5 as injection continues, and in Scenario 3 S_EFF_ persists at values greater than 0.5 over an extended period.

The extent to which these scenarios occur is of great significance with respect to long-term assessments of seismic hazard from hydraulic fracturing-induced seismicity. It is already established that in certain settings, runaway rupture has occurred, and as such we cannot use the McGarr cap as an upper bound for event magnitudes^10,28^. The extent to which runaway rupture can persist is also of significance. Hydraulic fracturing-induced seismicity hazard assessments are often performed using observed rates of seismicity per well^33^, with these rates extrapolated into the future without any consideration of whether the rates of seismicity observed during initial fault reactivation might be representative of longer-term behaviour. If the budget of tectonic strain energy is high relative to the amount released by hydraulic fracturing, then we might expect the rates of induced seismicity to persist at a high level, whereas if the budget of tectonic strain energy is significantly depleted by the induced seismicity, then we might expect rates of seismicity to decrease over time.

## Data and analysis

In this study, we compiled hydraulic-fracturing data available from provincial regulators (see “Methods and datasets” section) for wells in western Canada. We grouped wells into “blocks” of 0.2° in longitude by 0.1° in latitude (approximately 13 × 11 km), aggregating the total volume of fluid injected inside each HF well located inside each block. This area size was chosen to implement a similar spatiotemporal association filter proposed for HF-induced seismicity in western Canada in recent studies^8^, where only seismic events located less that 5 km from any well pad were associated with the HF stimulation of any unconventional well. This 5 km radius (or 10 × 10 km for gridded areas) was then rounded to the nearest 0.1 degree in geographical coordinates for simplicity. This spatial discretisation enables analysis of fluid volumes injected at the scale of well pads (i.e., multiple horizontal wells drilled at close distances from each other), instead of individual wells^8^, thereby recognising that wells from multiple different pads could influence the same fault structures. The block dimensions are also larger than typical location uncertainties of the seismic events reported in regional catalogs^34^. A sensitivity analysis of the block size is presented in our supplementary materials.

**Figure 4:**
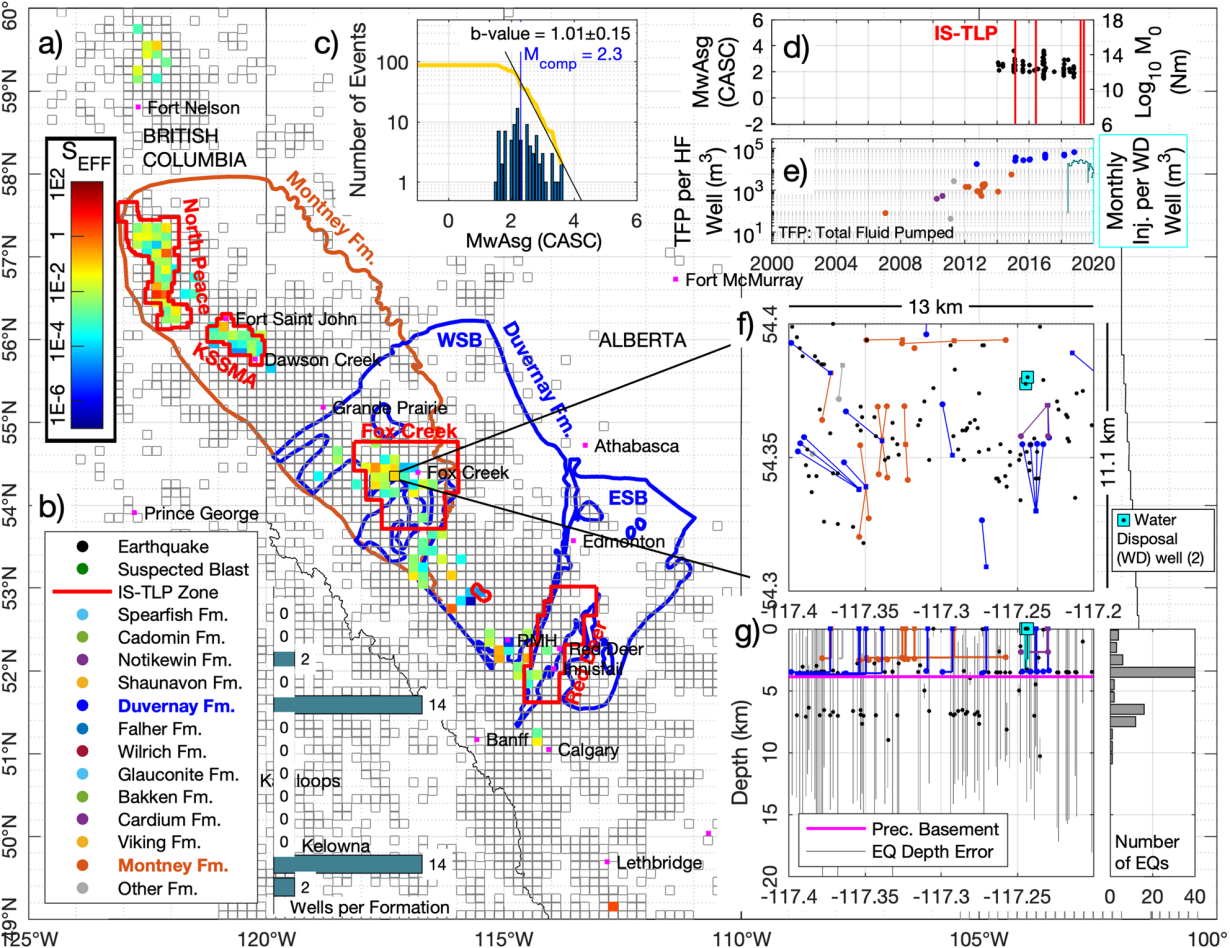
Example of the Seismic Efficiency Ratio (S_EFF_) calculated for one unit area of 0.2° in Longitude by 0.1° in Latitude (or approximately 13 × 11 km) near the town of Fox Creek in central Alberta. Most of the wells hydraulic fractured in this area were from the Duvernay and Montney formations (**b**), whereas the entire seismic activity in the same area (**d**) occurred only during the hydraulic stimulations of the Duvernay wells (**e**) despite all wells being located only a few kilometers apart from each other (shown in **f**). The shallow depth distribution of most of the same seismic events (shown in **g**), of less than 5 km, also suggests that these events where induced by the hydraulic fracturing stimulations of wells from the Duvernay formation (as natural events tend to have deeper locations). The seismic activity (**d**) has no clear correlation with the injection in the two water disposal (WD) wells (also shown in **e**) located inside the same unit area. The Gutenberg-Richter distribution of the seismic events from this area (shown in c), and calculated using a Maximum Likelihood Estimate method^39^), has a b-value close to 1 [as assumed in Eq. (2)], and a magnitude of completeness (Mcomp) of 2.3. The S_EFF_ calculated for all unit areas in western Canada, shown in the background map in a), is also shown in Fig. 6 together with the calculated b-value and observed maximum moment magnitude, and also in Video S2 and listed in Table S1 in the supplementary materials. All geoLOGIC systems ltd. data and software is copyright 2022.

To calculate S_EFF_ for each block, we compared injection volumes to the cumulative seismic moments of earthquakes recorded within the block (Fig. 4). To ensure that we only consider events that may have been induced by hydraulic fracturing, we only use events that occurred within 3 months after any hydraulic fracturing activity within a block. This temporal filter for HF-induced earthquakes has also been suggested by Schultz et al.^8^, and reflects maximum observed delay-times between injection and induced seismicity identified by Verdon and Bommer^30^ in their worldwide compilation of hydraulic fracturing induced seismicity case studies. Sequences of wastewater disposal induced seismicity have also been identified in the WCSB—such sequences are often easy to identify as persistent, long-standing clusters of seismicity^35^. Events within such sequences were removed from our analysis. Verdon and Bommer^36^ have demonstrated that only certain formations within the WCSB are susceptible to induced seismicity—for example, there are no reliably-documented cases of hydraulic fracturing induced seismicity from wells targeting shallow, Cretaceous-age formations in the WCSB. In our analysis, we only include wells that target formations below the base-Mannville unconformity, which is a major basin-wide stratigraphic feature of Lower Cretaceous age that marks the onset of clastic deposition in a foreland basin setting. By contrast, Palaeozoic and lower Mesozoic deposition in the WCSB took place on an extensional/transtensional passive margin^37^. Notwithstanding these steps, the assessment of whether an earthquake (or sequence of seismicity) is induced (and if induced, by what particular activity) is not trivial^38^, and so the inclusion of any particular event in our analysis does not mean that we explicitly assign causation to a particular activity.

**Figure 5:**
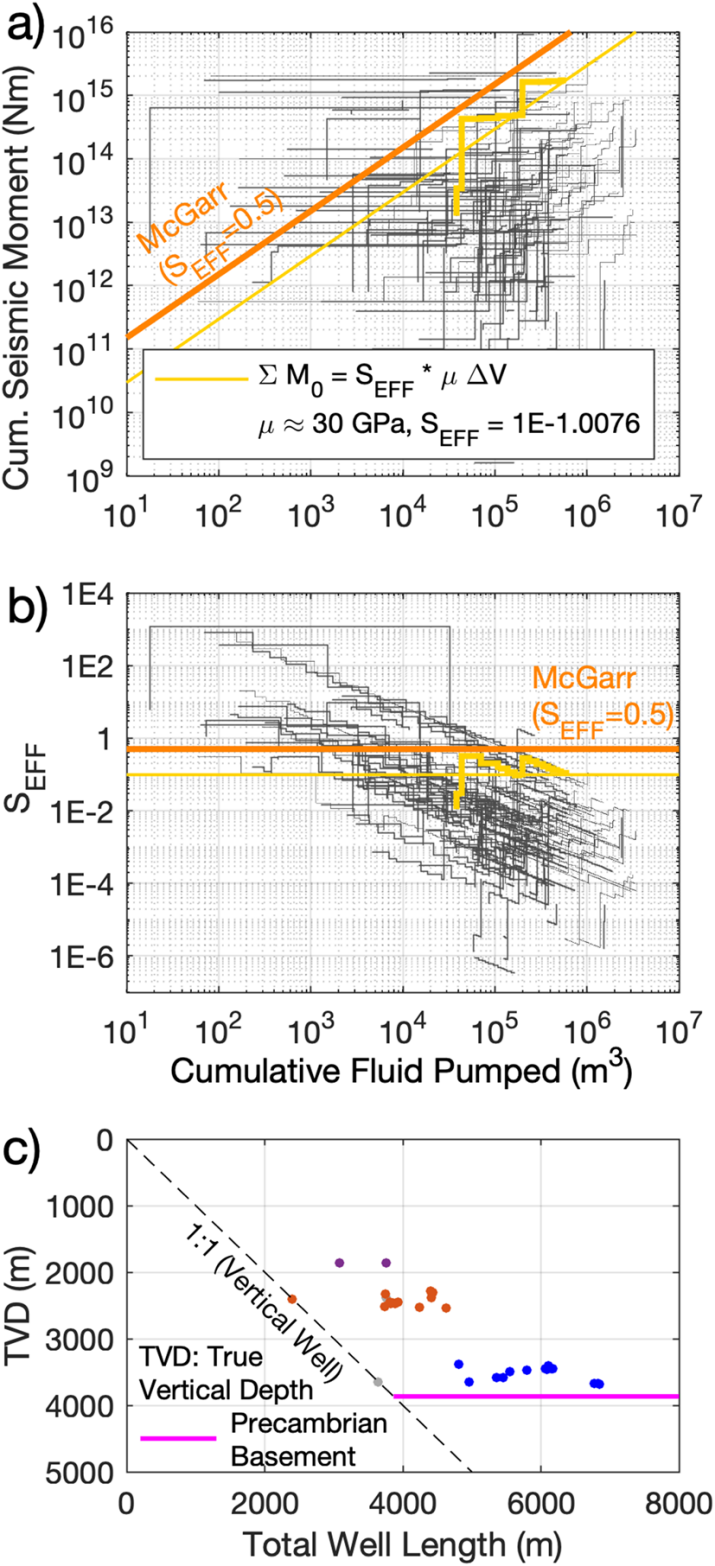
(**a**) Response path of the cumulative fluid pumped in all hydraulic-fractured wells located inside the unit areas of 0.2° in Longitude by 0.1° in Latitude shown in Fig. 4a (in grey), highlighting the response path of the unit area near Fox Creek obtained from the Total Fluid Pumped per well from this area (shown in Fig. 4e), and cumulative seismic moment of all seismic events inside the same area (shown in Fig. 4f near the HF wells, and their magnitudes shown in Fig. 4d). The response path calculated for all unit areas in western Canada, are also shown in Video S2 in the supplementary materials. The variability of the S_EFF_ for this example unit area near Fox Creek, shown in (**b**), shows two clear runaway rupture sequences (similar than the scenario 2 illustrated in Fig. 3b), attributable to the reactivation of two different (but relatively close) faults located inside this unit area. A constant S_EFF_ of 0.5, which corresponds to a seismic cycle with zero stress drop [from Eq. (4)] is also shown in (**a**) and (**b**) for reference. The True Vertical Depth (TVD) of the same hydraulic fractured wells are shown in (**c**), together with the TVD of the Precambrian basement (retrieved from the 3D provincial Geological Model of Alberta^48^). All geoLOGIC systems ltd. data and software is copyright 2022.

**Figure 6:**
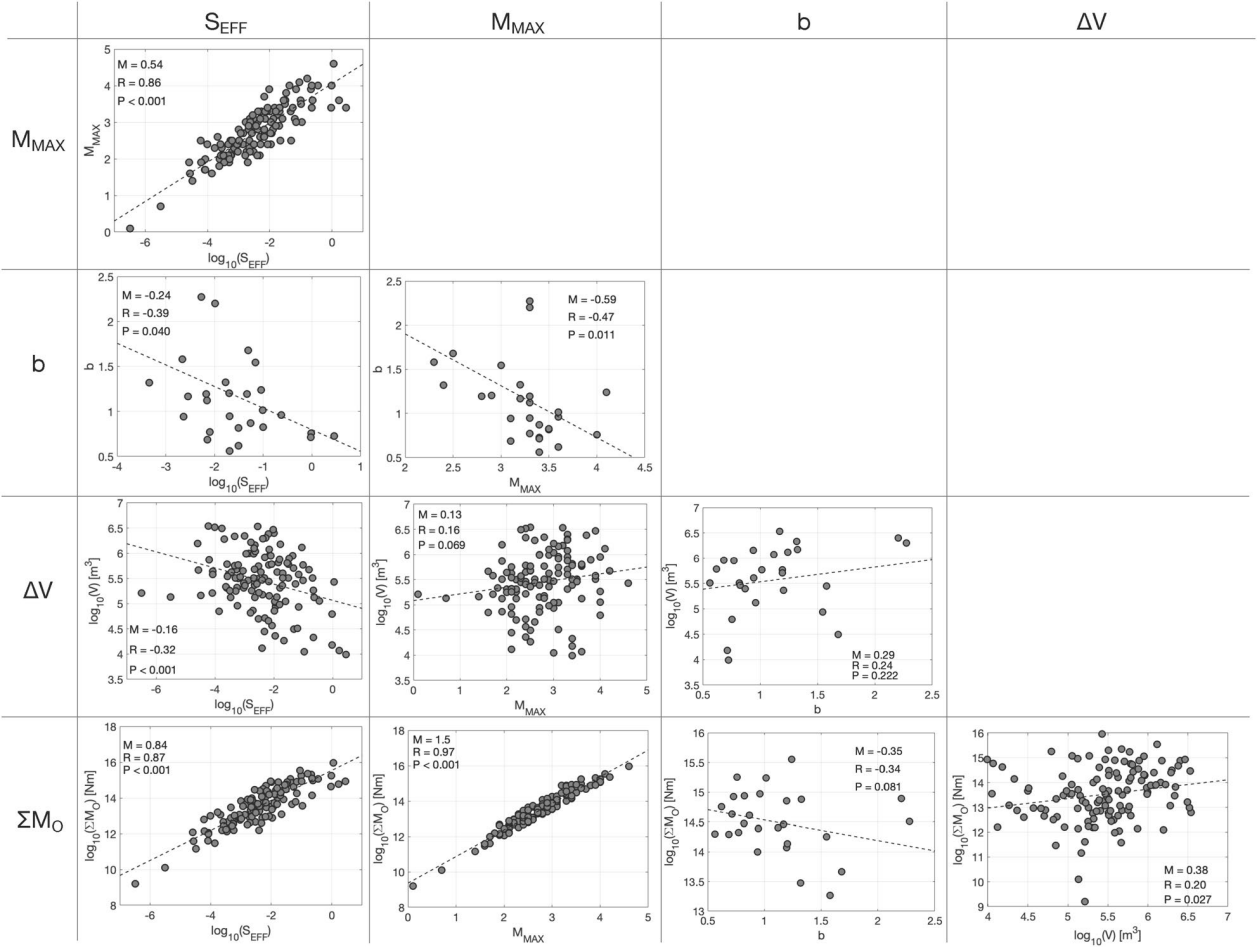
Cross-plots of variables for each grid block, including S_EFF_, M_MAX_, ΣM_0_, ΔV, and *b*-values. The dashed lines show the least-squares fit between each pair of variables. The gradient of this line, M, the Pearson’s correlation coefficient, R, and the statistical significance of this value, P, are reported in each sub-plot.

We computed the temporal evolution of S_EFF_ within each block (Fig. 5), in order to assess the extent to which each of the endmember scenarios described in Fig. 3 might apply to the induced seismicity within the WCSB. We also computed the *b*-value and magnitude of completeness of the Gutenberg-Righter distribution of the seismic events for each block with at least 20 seismic events (shown in Figs. 4, 6 and Video S2, and listed in Table S1 in the supplementary material). To calculate *b* values for each block, we first measured the catalog completeness (Mcomp in Fig. 4c) as the magnitude with the largest numbers of events, and then calculated the *a* and *b* values of the Gutenberg-Righter distribution (log_10_ *N* = *a* − *b*M, where N is the number of events with magnitudes greater than or equal to M) using the Maximum Likelihood Estimate method^39^ (see example in Fig. 4c).

## Results

Figure 5a,b show the evolution of S_EFF_ for all the blocks in our study area in which S_EFF_ is close to 0.5 at any point during the two decades covered by our study. We omit grid blocks where S_EFF_ ≪ 0.5, since these cases will not contribute to any assessment of the extent to which runaway rupture can occur and persist. We observe that values of S_EFF_ higher than 0.5 are evident, indicating that runaway rupture has likely occurred. We identified 14 such cases, which are shown in detail in the Supplementary Material. However, the general trend for these cases is for S_EFF_ to decrease to < 0.5 as injection proceeds, in accordance with Scenario 2 of Fig. 3. Indeed, there are no cases for which S_EFF_ is greater than 0.5 after the cumulative injection of more than 10^5^ m^3^. Given that typical injection volumes for Montney, Horn River and Duvernay wells are 20,000 m^3^ or more, this would indicate that runaway rupture does not persist after stimulation of more than 4 or 5 wells within a given block.

In Fig. 6, we provide cross-plots of relevant variables for each grid block, including ΣM_0_, S_EFF_, the cumulative injection volume ΔV, the largest observed magnitude M_MAX_, and *b*-values. Figure 6 also shows the gradient, *m*, of the least-squares fit between each pair of variables (for ΣM_0_, S_EFF_, and ΔV, fitted in log space), the Pearson’s correlation coefficient, *R*, and the *P* values for assessing the null hypothesis that there is no relationship between the variables. Some of the observed correlations are trivial and expected, such as between S_EFF_ and ΣM_0_ (as per Eq. 3), and between M_MAX_ and ΣM_0_.

We note the statistically significant negative correlation between S_EFF_ and ΔV: this supports the situation discussed in the previous paragraph where high S_EFF_ values do not persist as larger volumes are injected, leading to a negative correlation. We note the absence of correlation between ΔV and M_MAX_: this is because the occurrence of large events is primarily controlled by the presence of high S_EFF_ values, rather than high injection volumes. As a result, we observed strongly significant correlation between M_MAX_ and S_EFF_. We observe negative correlation between *b*-values and S_EFF_: high S_EFF_ values (S_EFF_ > − 1) are universally associated with *b*-values of approximately 1.0 or less, equivalent to values commonly observed for tectonic earthquakes^40^. In contrast, many of the blocks with lower S_EFF_ values are associated with higher *b*-values. This is consistent with the hypothesis that cases with high S_EFF_ represent situations where tectonic stress is released, and hence the *b*-values are similar to those observed for tectonic earthquakes. In contrast, higher *b*-values are often argued to indicate seismicity driven by fluid-movement within fault and fracture networks^41^, and hence indicative of seismicity driven directly by fluid injection rather than the release of tectonic stress. Overall, the strong correlations between the rates and magnitudes of the induced seismicity and the S_EFF_ value shows that, for improved seismic hazard assessment, it is vital that we understand the controls on seismic efficiency, and how it might vary over both space and time for a given target formation and type of industrial activity. Understanding the controls on S_EFF_ are more important the *b* values, which tend to revert to tectonic values once large earthquakes, driven by release of tectonic stresses, begin to occur.

## Discussion

Figures 4 and 5 show an example of the temporal evolution of S_EFF_ calculated for one unit block near the town of Fox Creek in central Alberta. A similar plot of the S_EFF_ for each unit block is shown in Video S2 in the Supplementary Material. The response path of this area, as delineated by the evolution of S_EFF_, shows evidence for runaway rupture with an initial sharp increase in the cumulative seismic moment. Over time, however, while seismic activity continues, it does so at a more gradual rate, resulting in a decrease in the S_EFF_, implying that seismicity becomes dominated by arrested ruptures that release strain energy imparted by the injection process. Figures S5 to S18 in the Supplementary Material highlights the unit blocks from Video S2 that had runaway ruptures (i.e., areas with S_EFF_ higher than 0.5 at any moment).

Kao et al.^42^ calculated the rate of tectonic moment accumulation across the WCSB, based on geodetic observations of tectonic strain. They found that injection induced seismicity typically occurred in areas where the tectonic moment rate was M_O_^TS^ = 1–2 × 10^6^ Nm/km^2^/yr. Adjusting this rate for our blocks with areas of 11 × 13 km gives a tectonic moment rate of M_O_^TS^ = 1.43–2.86 × 10^8^ Nm/block/yr. On this basis, the occurrence of a single M 3.0 induced earthquake within a grid block, if it primarily releases accumulated tectonic strain energy, represents the tectonic strain accumulated over 100,000 years. This implies that the tectonic strain rate in the WCSB is insufficient to reload a fault and thus allow continued occurrence of runaway rupture within a given area that is subject to repeated injection activities. This observation is supported by our compilation of S_EFF_ values within the basin: runaway rupture, as indicated by larger magnitude events occurring after small injection volumes, giving S_EFF_ > 0.5, can (and does) occur. However, S_EFF_ > 0.5 does not appear to persist as injection continues in a given location, which is consistent with the fact that a handful of induced events of moderate magnitude may be sufficient to release the tectonic strain accumulated over many thousands, or indeed millions of years.

**Figure 7:**
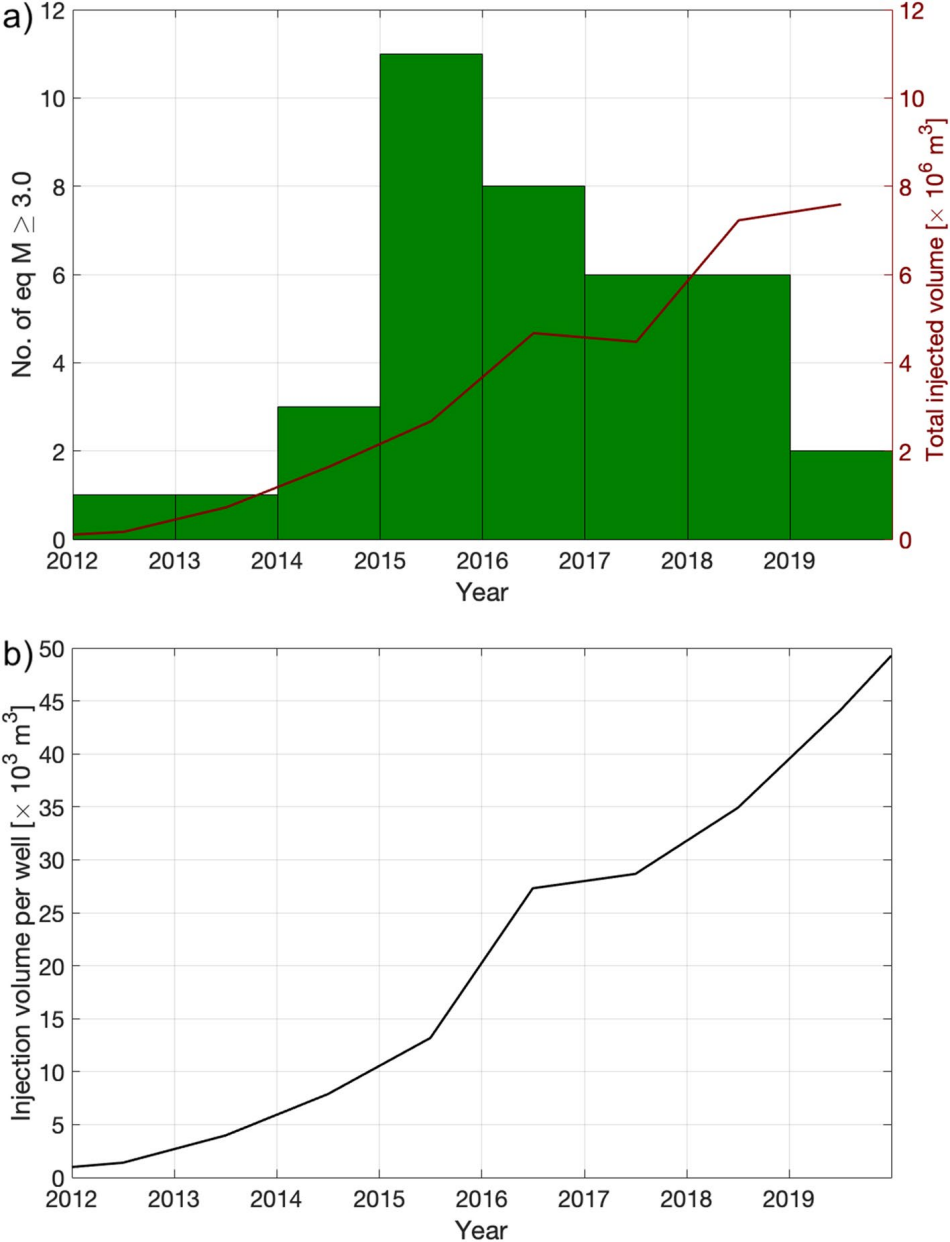
(**a**) Number of earthquakes of magnitude ≥ 3.0 per year reported inside the Fox Creek area designated in the Alberta Energy Regulator’s Subsurface Order No. 2 (SSO2)^16^ (shown in Figs. 1a and 4a), and total volume injected in HF wells inside the same area. (**b**) Average injected volume per year per HF well inside the Fox Creek area. Note the gradual decrease in the number of earthquakes per year of magnitude ≥ 3.0 since 2015, despite the constant increase in the total injected volume per year in HF wells, and in the average injected volume per HF well.

In the Fox Creek area in central Alberta, Reyes-Canales et al.^43^ performed an assessment of event rates and Gutenberg-Richter *b*-values. This area is the subject of the Alberta Energy Regulator’s Subsurface Order No. 2 (SSO2)^16^, imposing a Traffic Light Protocol with a red light of M 4.0. The rate of occurrence of M > 3.0 events in this area shows a gradual decrease from 2015 through to 2020, while injection volumes into the Duvernay within this area have continued to rise (Fig. 7a). Reyes-Canales et al.^43^ suggested that some of this reduction in seismicity could be attributed to the targeting areas less susceptible to induced seismicity. However, it is also clear that there has been a reduction in the seismic activity of the eastern region inside the Fox Creek area that previously exhibited higher seismic activity. This reduction in the seismic activity occurred despite the per-well injection volumes in this area continuing to rise during this time (Fig. 7b). Reyes-Canales et al.^40^ suggested that the reduction could have been driven by the implementation of the SSO2 TLP, which encourages the operators to exercise additional precautions and mitigation strategies to avoid induced seismicity.

Our results presented above suggest an alternative hypothesis to explain the reduction in seismicity seen in the Fox Creek region since 2015. Rather than being primarily driven by the introduction of the TLP, it instead may represent a situation in which runaway rupture has become less prevalent as the tectonic strain accumulated over millennia is released by initial hydraulic fracturing in the area, with later wells subsequently limited by the the moment cap indicated by Eq. (3) with respect to the maximum available moment that could be generated. The relative significance of these factors could be further addressed by examining in more detail the extent to which operators have successfully taken pro-active steps to mitigate induced seismicity in practice—no actual examples of such were presented by Reyes-Canales et al.^40^, although detailed information of operators’ mitigation steps is seldom publicly available. Even in cases where seismogenic faults have been identified, mitigation steps taken by operators have often not been effective^28,44^. The fact that per-well injection volumes have continued to increase indicates that the TLP has not led to any major changes in operators’ completion strategies. Moreover, the reduction in seismicity in the Fox Creek area is observed at all magnitudes, whereas the SSO2 TLP, with a red light at M 4.0, is only designed to mitigate larger-magnitude events.

## Conclusions

The WCSB has a wide variety of geological and tectonic features that influence the seismic response of unconventional wells to hydraulic fracturing stimulations. Responses range from virtually zero seismicity near shallower Bakken and Viking wells located in the east side of the basin, to the seismically active areas near some longer and deeper Duvernay and Montney wells on the west side of the basin. In some cases, the maximum magnitude has surpassed the expected levels estimated with McGarr’s cap function, which is commonly used to estimate the maximum seismicity level associated with fluid-injection operations. We fit a seismic efficiency ratio (S_EFF_), the ratio of the net seismic moment release and the forecasted maximum moment, and find that the obtained S_EFF_ exhibits a complex evolution in such areas, with anomalously high seismic activity arising from inferred runaway rupture processes on pre-existing faults. In 93% of cases where exceedance of S_EFF_ = 0.5 occurred (i.e., in 13 out of 14 cases), representing the presumed onset of stored tectonic stress release, continued injections within the same 0.2° × 0.1° area (in Latitude x Longitude, of approximately 13 × 11 km) did not lead to further seismicity with characteristics of runaway rupture.

## Methods and datasets

To study the recent seismicity associated with hydraulic fracturing operations in the WCSB, we first compiled public seismic catalogs from the Composite Alberta Seismicity Catalog (CASC, retrieved from https://www.inducedseismicity.ca/catalogues/, last accessed on August 2021) that includes all seismic events reported in Alberta and NE British Columbia until January 2020, in the seismic catalog from the Geological Survey of Canada (GSC), Alberta Geological Survey (AGS), US Geological Survey (USGS), and the TransAlta/Nanometrics seismic network installed in 2013 around the Brazeau Dam in west-central Alberta^45^. The CASC catalog first eliminates duplicate events that appear in different catalogs, and classifies each event as an earthquake or a suspected blast (discriminated from seismic events from its time of occurrence -as every mine blast is schedule in afternoon hours only to ensure plenty of daylight during each blast– and its epicenter proximity to open pit coal mines or quarries). The AGS also provides in their seismic catalog (publicly available from https://ags-aer.shinyapps.io/Seismicity_waveform_app/) a similar discrimination for each reported seismic event, as Suspected Earthquake (SE), Suspected Induced (SI) or Known Induced (KI), although for KI events it does not specify induced by what (i.e., hydrocarbon production, enhanced oil recovery, salt water disposal, or hydraulic fracturing, all of them previously reported in western Canada^46^).

The CASC catalog also assigns a moment magnitude (MwAsg) to each event to normalize the magnitudes reported in different catalogs and using different scales (mostly local magnitude)^45^. The seismic events in the catalogs from the Horn River Basin^47^ and the GSC for the provinces of Saskatchewan and Manitoba were also added to the CASC catalog to cover the seismicity reported in the entire basin between 2000 and 2020.

The stimulation data from the wells hydraulic fractured in the same period (as injected fluid volumes, target formation, and well depth, length, and orientation) was retrieved with the software geoSCOUT (see Data availability). We then clustered the earthquake and well data in unit areas of 0.2° in Longitude by 0.1° in Latitude (or approximately 13 × 11 km, somewhat larger than a standard township of 6 × 6 miles) to calibrate McGarr’s cap function (Eq. 2) to estimate the maximum magnitude of an induced seismic event by calculating for each unit area a Seismic Efficiency Ratio (S_EFF_) from the total fluid pumped in every hydraulic fractured well, and the cumulative seismic moment from every seismic event reported inside each unit area. In the case of Alberta, we were also able to compare the wells’ proximity to the basement, as shown in Figs. 4g and 5c (another key geological parameter in relation to seismicity induced by water injection operations including hydraulic fracturing) from the depth of the Precambrian basement included in the province’s 3D geological model^48^. This was not possible for other unit areas within the same WCSB, particularly in NE British Columbia (as shown in Video S2 in the supplementary material), where no detailed map of the Precambrian basement has been released to date. Finally, the location of the water disposal wells, as the ones shown in Fig. 4f, were retrieved from the public databases of the provincial regulators of Alberta and British Columbia, and their monthly injection data is also publicly available in https://petroninja.com/ (see more details in Data availability). The authors thank geoLOGIC systems ltd. for their contribution of data and software used in this study. All geoLOGIC systems ltd. data and software is copyright 2022.

### Additional information

The supplementary material contains two animations (Videos S1 and S2) and one table (Table S1) based on Figs. 1 and 4, showing the temporal and spatial variation of hydraulic fracturing and seismic activity in the WCSB between the years 2000 and 2020. A sensitivity analysis of grid areas of different sizes and locations used for the estimation of the Seismic Efficiency Ratio (S_EFF_) inside each area, and their response path and variability of S_EFF_ overtime (based on Fig. 5), are shown in Figs. S1 to S4, and the 14 cases of runaway rupture observed in our study are shown in detail in Figs. S5 to S18.

## Supplementary Information

Supplementary Information 1.

Supplementary Video 1.

Supplementary Video 2.

Supplementary Information 4.

Supplementary Information 5.

Supplementary Information 6.

## Data Availability

The regional seismic catalogs from western Canada used in this study are publicly available (see “Methods and datasets”). For convenience, the well information from the hydraulic fractured wells in the WCSB was retrieved using geoSCOUT software from geoLOGIC Systems Ltd., licensed to the Microseismic Industry Consortium, University of Calgary. Information from the HF stimulation from the same wells is also publicly available in http://fracfocus.ca/en and in the public databases of the provincial regulators of Alberta and British Columbia (https://www1.aer.ca/ProductCatalogue/WELL.html; https://reports.bcogc.ca/ogc/app001/r/ams_reports/1), that also include the location of the water disposal (WD) wells in both provinces. The monthly injection data of the WD wells reproduced in this manuscript and in its supplementary material is also publicly available from https://petroninja.com/.
