# Supplementary material for: Basin-scale multi-decadal analysis of hydraulic fracturing and seismicity in western Canada shows non-recurrence of induced runaway fault rupture
Source: Sci Rep. 2022 Aug 24;12:14463. doi: 10.1038/s41598-022-18505-0 (PMC9402563; doi:10.1038/s41598-022-18505-0)
Supplement: Supplementary file 1 — Supplementary Information 1. [file 41598_2022_18505_MOESM1_ESM.docx]

**Supplementary Material**

**Title: Basin-scale analysis of injection data and seismicity shows that, in western Canada, runaway induced earthquakes do not recur within a multi-year timescale on the same fault**

Germán Rodríguez-Pradilla ^a^, David W. Eaton ^b^ and James Verdon ^c^

^a^ Formerly at Department of Geoscience, University of Calgary. Calgary, Alberta, Canada. Now at School of Earth Sciences, University of Bristol. Bristol, United Kingdom

^b^ Department of Geoscience, University of Calgary. Calgary, Alberta, Canada

^c^ School of Earth Sciences, University of Bristol. Bristol, United Kingdom

This supplementary material provides: animations showing the temporal evolution of hydraulic fracturing and induced seismicity in the WCSB; data tables of the variables computed for each grid block in our analysis; a sensitivity analysis of the effect of the grid origin and dimensions; and further details of the 14 grid blocks where high S_EFF_ values have been observed.

**Animations and Data for each grid block**

**Video S1** shows the temporal variation of the seismic and hydraulic fracturing activity in western Canada between 2000 and 2020, as described in Figures 1 and 2 of the main manuscript. All geoLOGIC systems ltd. data and software is copyright 2022.

**Video S2** shows S_EFF_ calculated for each unit area of 0.2° longitude by 0.1° latitude in the WCSB, as described in Figures 4 and 5 of the main manuscript. All geoLOGIC systems ltd. data and software is copyright 2022.

**Table S1** provides the computed variables for seismic activity and hydraulic fracturing for each grid block (0.2° longitude by 0.1° latitude). These parameters are used to calculate the Seismic Efficiency Ratio (S_EFF_). Column headers are: **Lon**, **Lat** (central coordinates of each grid block), **NumEQs** (number of earthquakes), **CumM0_Nm** (Cumulative seismic moment, $\sum M_{0}$ , in Nm), **MaxMw**, **MinMw** (maximum and minimum observed moment magnitudes), **a_value_MLE**, ***b*-value_MLE, db_MLE,** (a-value, b-value, and the b-value’s standard deviation, from the Gutenberg-Richter distribution of the seismic events reported inside each block calculated using a Maximum Likelihood Estimation method [1]; only calculated for areas with at least 20 seismic events, *b*-value = 0 means not enough seismic events to calculate), **Mcomp** (catalog completeness from the magnitude distribution of seismic events), **NumHFWells** (number of hydraulic-fractured wells), **CumTPF_m3** (Cumulative Total Fluid Pumped in HF, in m3), **S_EFF_** (Seismic Efficiency Ratio), **Aphi** ($A_{\phi}$, relative stress magnitudes), **SHmax_Az** (azimuth of the maximum horizontal stress), **NumDispWells** (number of water disposal wells), **UWI_DispWells** (Unique Well Identifier of each water disposal well inside each unit area). The regional stress parameters, Aphi and SHmax_Az, were retrieved from Lund Snee and Zoback [2]. All geoLOGIC systems ltd. data and software is copyright 2022.

**Grid size and origin sensitivity analysis**

The analysis presented in our main text is based on cumulative seismic moments and injection volumes summed within discrete grid blocks with dimensions of 0.2° longitude by 0.1°. This choice of dimension was based on considerations of hydraulic fracturing length, location uncertainties, and the fact that multiple wells could, in theory, reactivate the same feature. Nevertheless, we have conducted a sensitivity analysis to assess the impact of larger or smaller grid sizes, as well as shifting the grid origin to evaluate whether any observations were artefacts created by arbitrary positioning of grid boundaries.

**Figure S1** shows the S_EFF_ calculated for unit areas of 0.2° in Longitude by 0.1° in Latitude (or approximately 13x11km) in the WCSB, with the inset showing as an example a zoomed view of one unit area near the town of Fox Creek, Alberta (same as Figures 4a and 4f from the main manuscript). The inset from this figure shows the impact of choosing a grid dimension of 0.1° x 0.05°: given typical lengths of horizontal HF wells, a significant portion of wells have their injection volumes split between grid cells. We suggest that a smaller gridding is therefor inappropriate.

**Figure S2** shows the S_EFF_ calculated for larger unit areas of 0.4° in Longitude by 0.2° in Latitude, or approximately 26x22km. This results in a lower resolution (when compared with the 13x11 km areas from Figure 4), as areas with high seismic activity and S_EFF_ may be averaged with adjacent areas that have lower S_EFF_.

**Figure S3** shows the S_EFF_ calculated for unit areas of 0.2° in Longitude by 0.1° in Latitude, shifted by half-size of each unit area (i.e., 0.1° in Longitude by 0.05° in Latitude) from the areas shown in Figure 4.

**Figure S4** shows the results produced by the grids shown in Figures S2 – S3. In (a) we show the response paths (ΣM_O_ vs ΔV), and in (b) we show the evolution of S_EFF_ for the 0.2° x 0.1° grid blocks as per Figure 4. In (c) and (d) we show the equivalent response for the larger 0.4° x 0.2° blocks shown in Figure S2, and in (e) and (f) we show the equivalent response for the shifted 0.2° x 0.1° grid blocks shown in Figure S3. In each case, we observe behaviour that is consistent with the observations presented in the main article: that in some grid blocks S_EFF_ values are initially high, but these values decrease as injection persists.

**Runaway rupture cases**

**Figures S5 to S18** show the 14 grid blocks in which runaway rupture, i.e., areas with S_EFF_ higher than 0.5 at any moment, has been observed. The format of these figures is described in Figures 4 and 5. All geoLOGIC systems ltd. data and software is copyright 2022.

# References of the Supplementary Material

| [1] | K. Aki, "Maximum likelihood estimate of b in the formula logN=a-bM and its confidence limits.," *Bulletin of the Earthquake Research Institute,* vol. 43, p. 237–239, 1965. |
| --- | --- |
| [2] | J. E. Lund Snee and M. D. Zoback, "Multiscale variations of the crustal stress field throughout North America," *Nature Communications,* vol. 11, no. 1951, 2020. |
| [3] | R. Schultz, G. Atkinson, D. W. Eaton, Y. J. Gu and H. Kao, "Hydraulic fracturing volume is associated with induced earthquake productivity in the Duvernay play," *Science,* vol. 359, pp. 304-308, 2018. |
| [4] | R. Schultz, V. Stern, Y. J. Gu and D. Eaton, "Detection Threshold and Location Resolution of the Alberta Geological Survey Earthquake Catalogue," *Seismological Research Letters,* vol. 86, no. 2A, p. 385–397. https://doi.org/10.1785/0220140203, 2015. |
