# Supplementary figures and images for: Basin-scale multi-decadal analysis of hydraulic fracturing and seismicity in western Canada shows non-recurrence of induced runaway fault rupture
Source: Sci Rep. 2022 Aug 24;12:14463. doi: 10.1038/s41598-022-18505-0 (PMC9402563; doi:10.1038/s41598-022-18505-0)

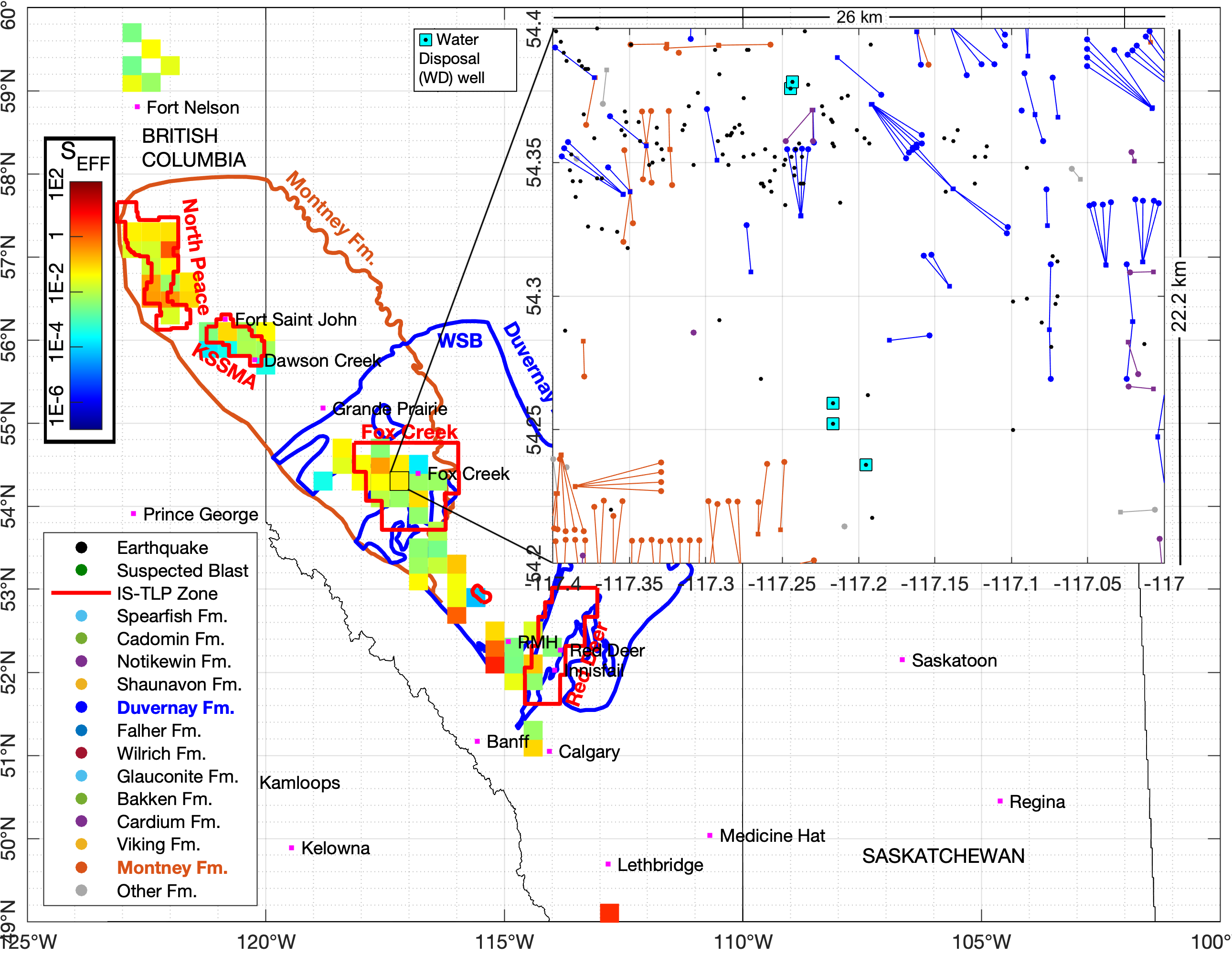

Supplement: Supplementary file 5 — Supplementary Information 5. [file 41598_2022_18505_MOESM5_ESM.zip › Figure S1 to S4/Figure S2 - Block size 0.4Lon x 0.2Lat.png]

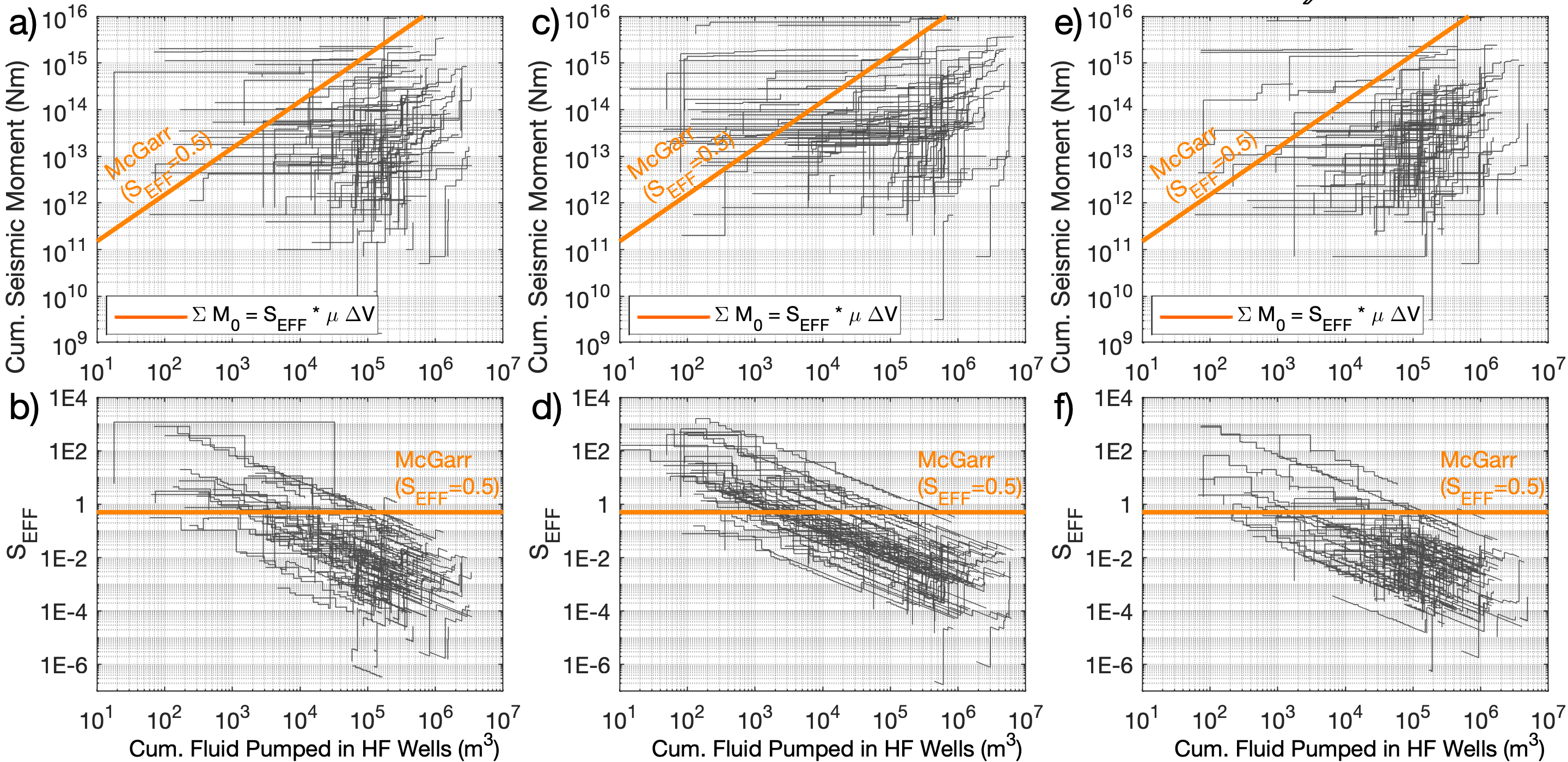

Supplement: Supplementary file 5 — Supplementary Information 5. [file 41598_2022_18505_MOESM5_ESM.zip › Figure S1 to S4/Figure S4 - Response Paths for Different Block Sizes.png]

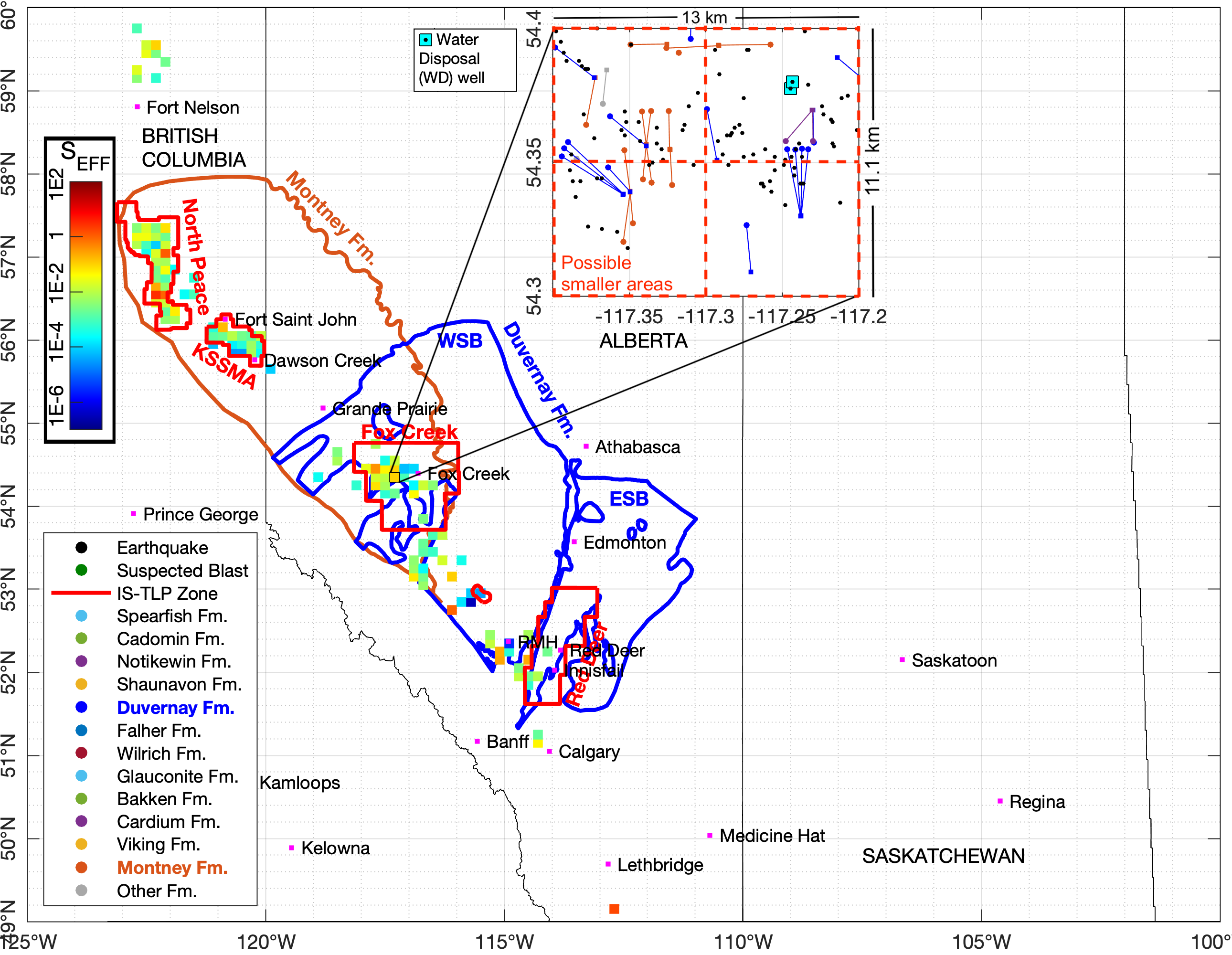

Supplement: Supplementary file 5 — Supplementary Information 5. [file 41598_2022_18505_MOESM5_ESM.zip › Figure S1 to S4/Figure S1 - Block size 0.2Lon x 0.1Lat+PossibleSmallerAreas.png]

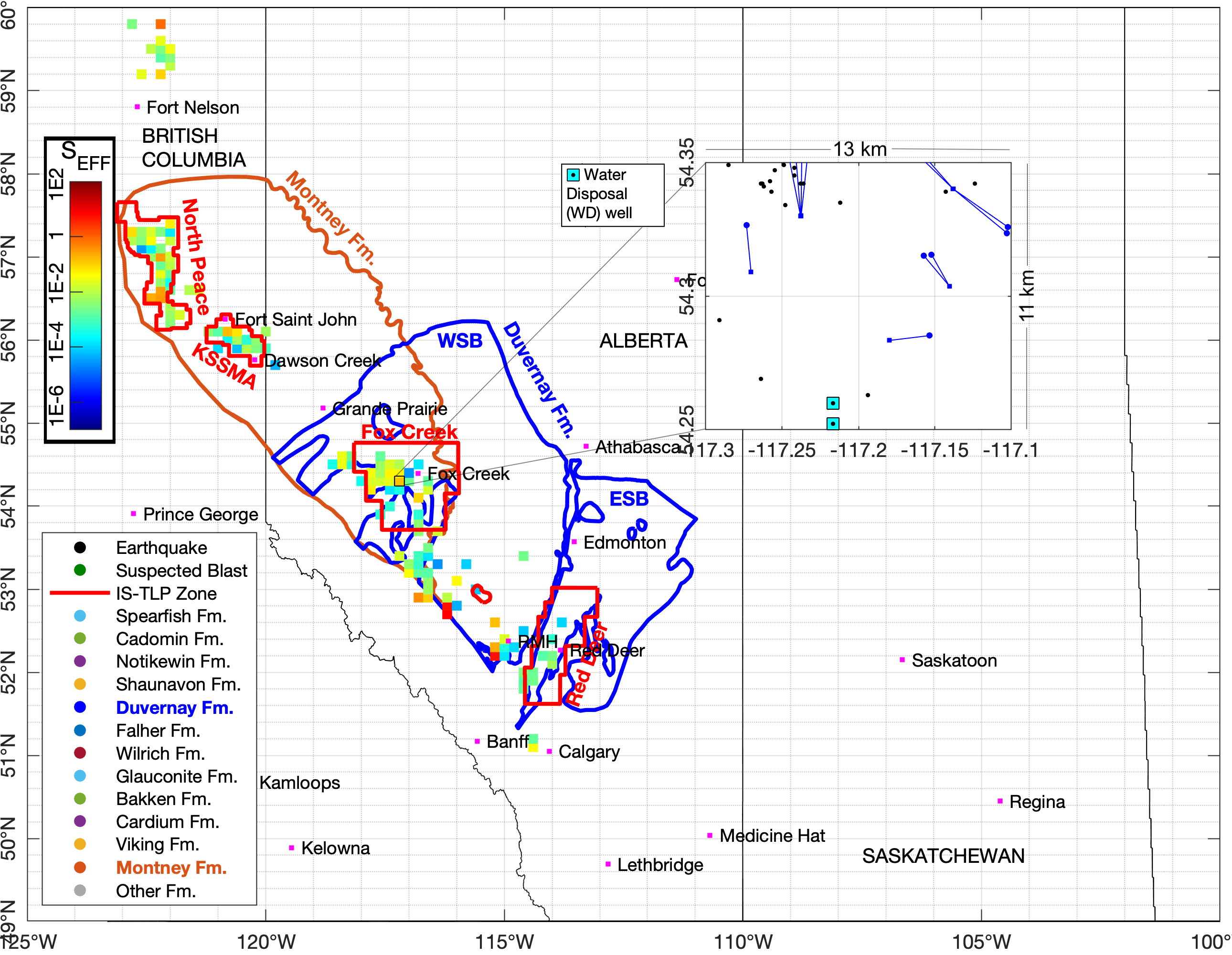

Supplement: Supplementary file 5 — Supplementary Information 5. [file 41598_2022_18505_MOESM5_ESM.zip › Figure S1 to S4/Figure S3 - Shifted Block size 0.2Lon x 0.1Lat.png]

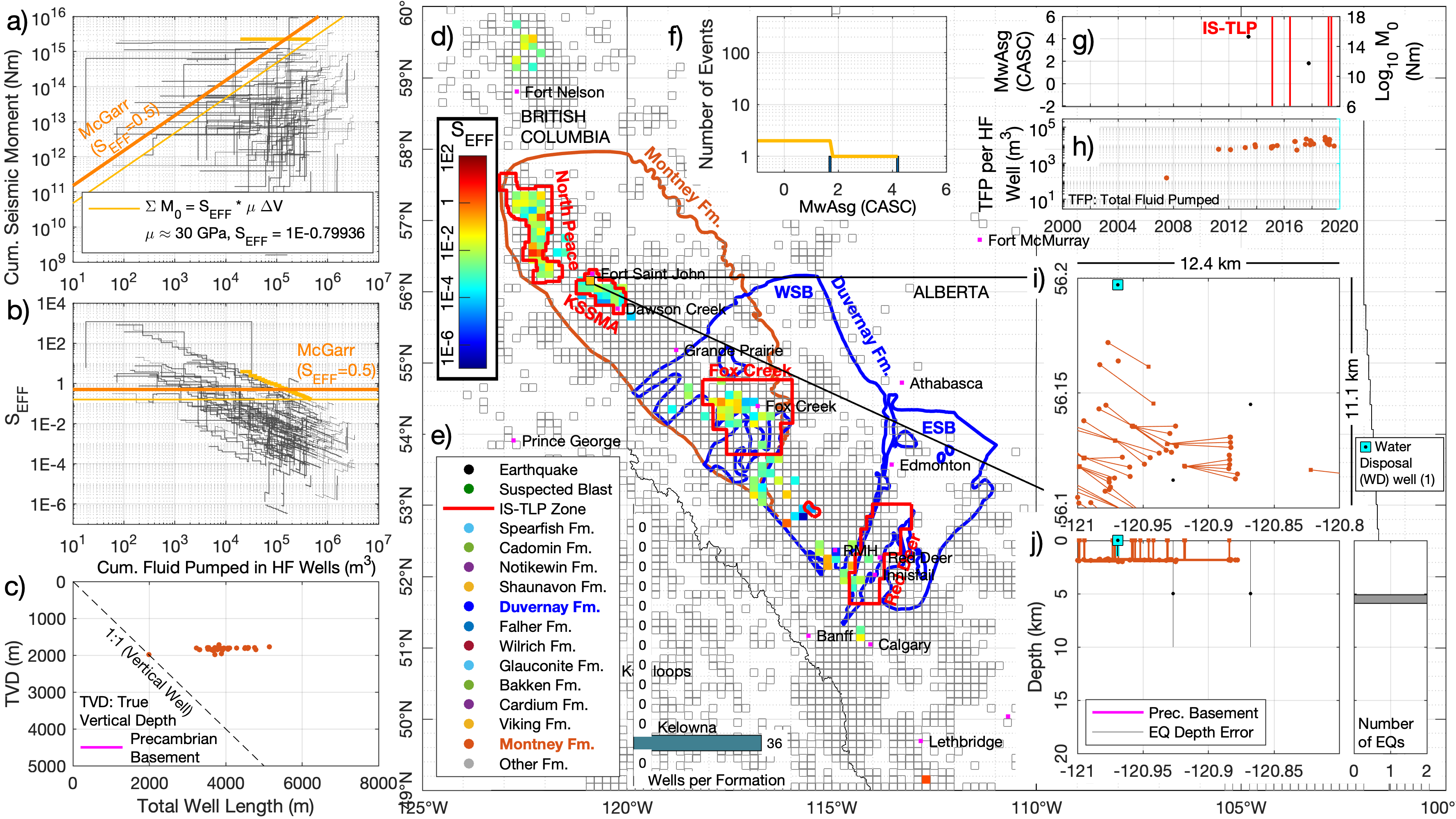

Supplement: Supplementary file 6 — Supplementary Information 6. [file 41598_2022_18505_MOESM6_ESM.zip › Figure S5 to S18 - 14 cases of runaway rupture/Figure S8 - AllFracs_CumTFP_CumM0_VariableSeff_Map_Cell_45.png]

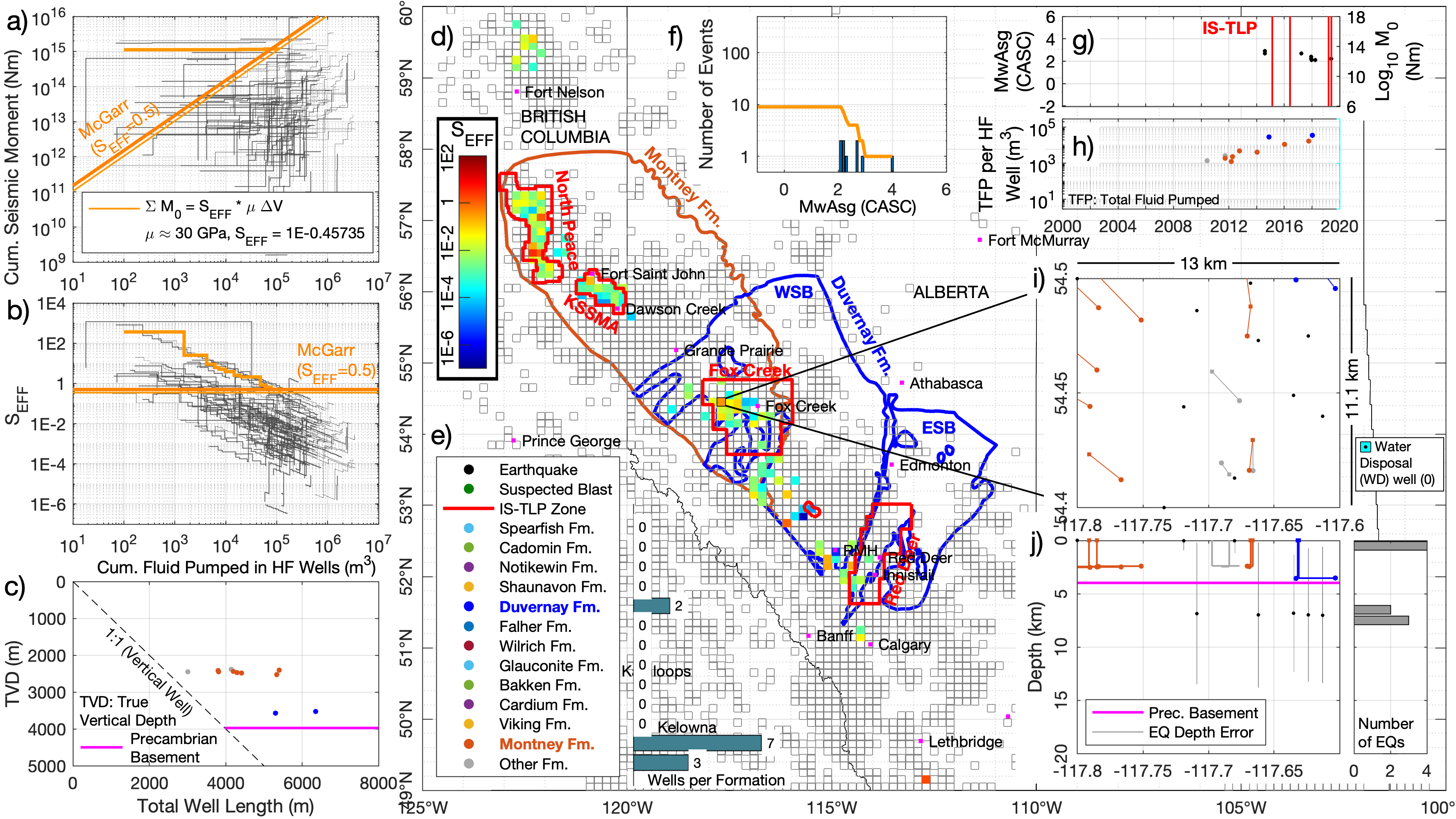

Supplement: Supplementary file 6 — Supplementary Information 6. [file 41598_2022_18505_MOESM6_ESM.zip › Figure S5 to S18 - 14 cases of runaway rupture/Figure S9 - AllFracs_CumTFP_CumM0_VariableSeff_Map_Cell_72.png]

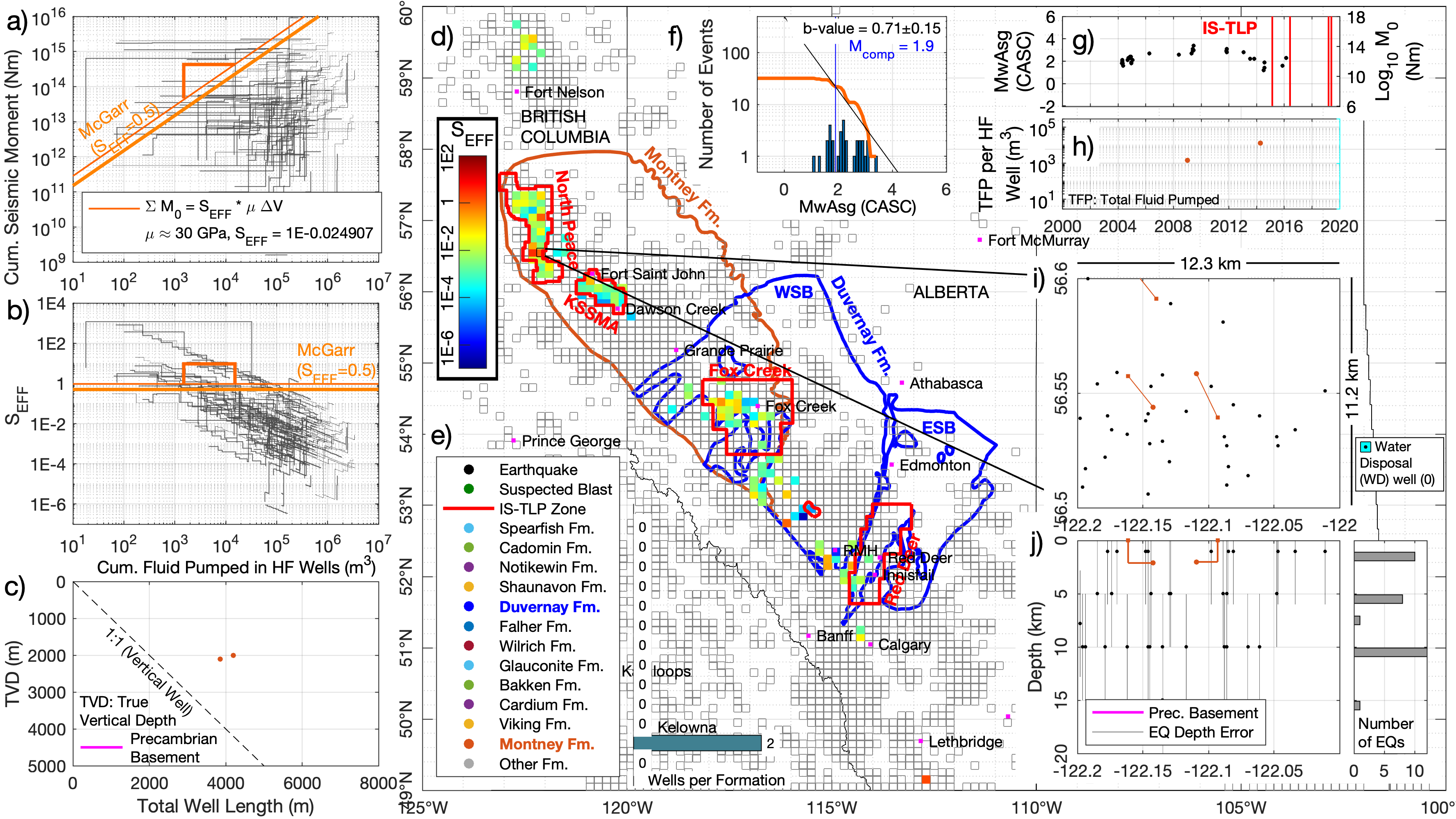

Supplement: Supplementary file 6 — Supplementary Information 6. [file 41598_2022_18505_MOESM6_ESM.zip › Figure S5 to S18 - 14 cases of runaway rupture/Figure S6 - AllFracs_CumTFP_CumM0_VariableSeff_Map_Cell_37.png]

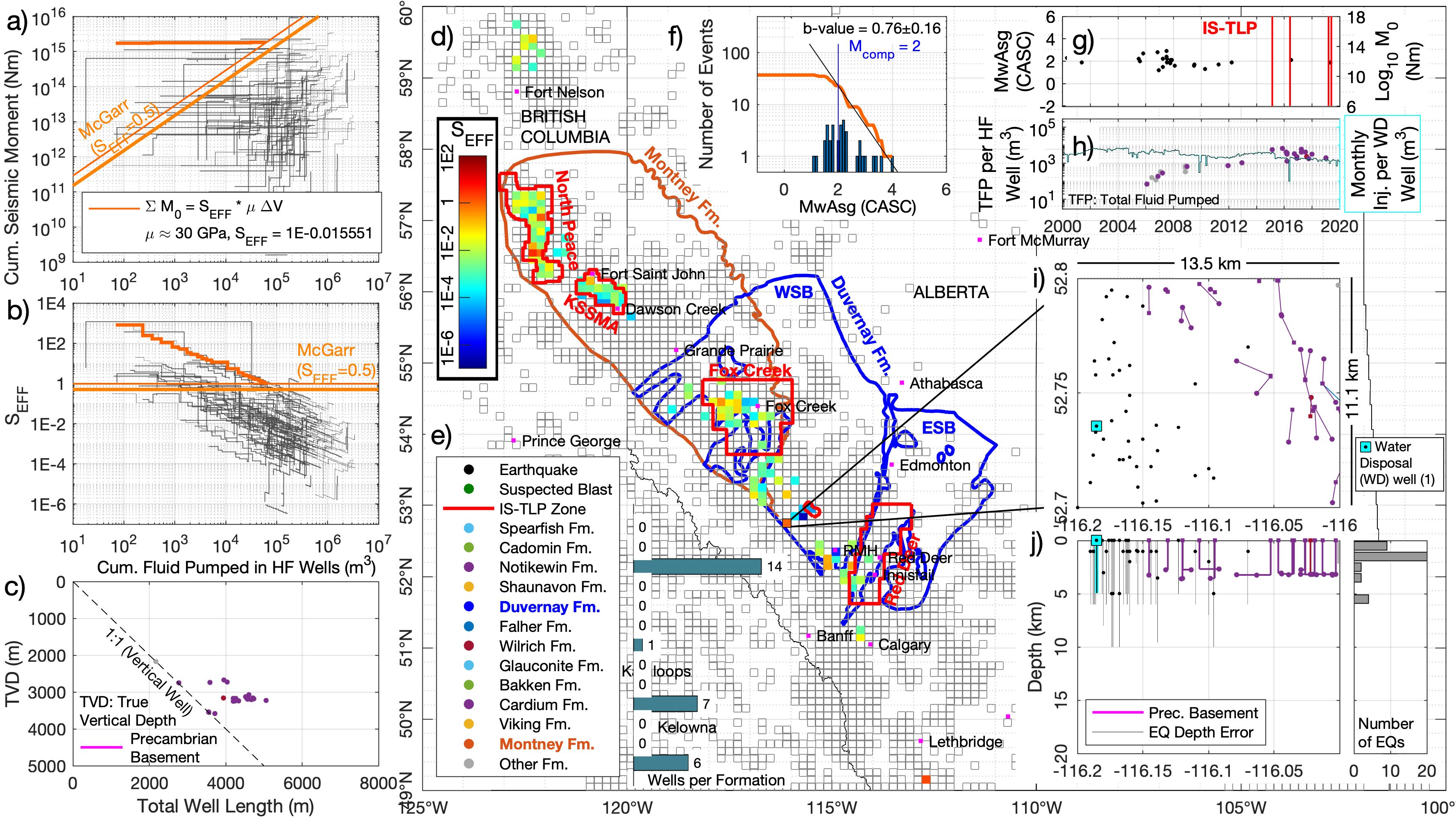

Supplement: Supplementary file 6 — Supplementary Information 6. [file 41598_2022_18505_MOESM6_ESM.zip › Figure S5 to S18 - 14 cases of runaway rupture/Figure S14 - AllFracs_CumTFP_CumM0_VariableSeff_Map_Cell_107.png]

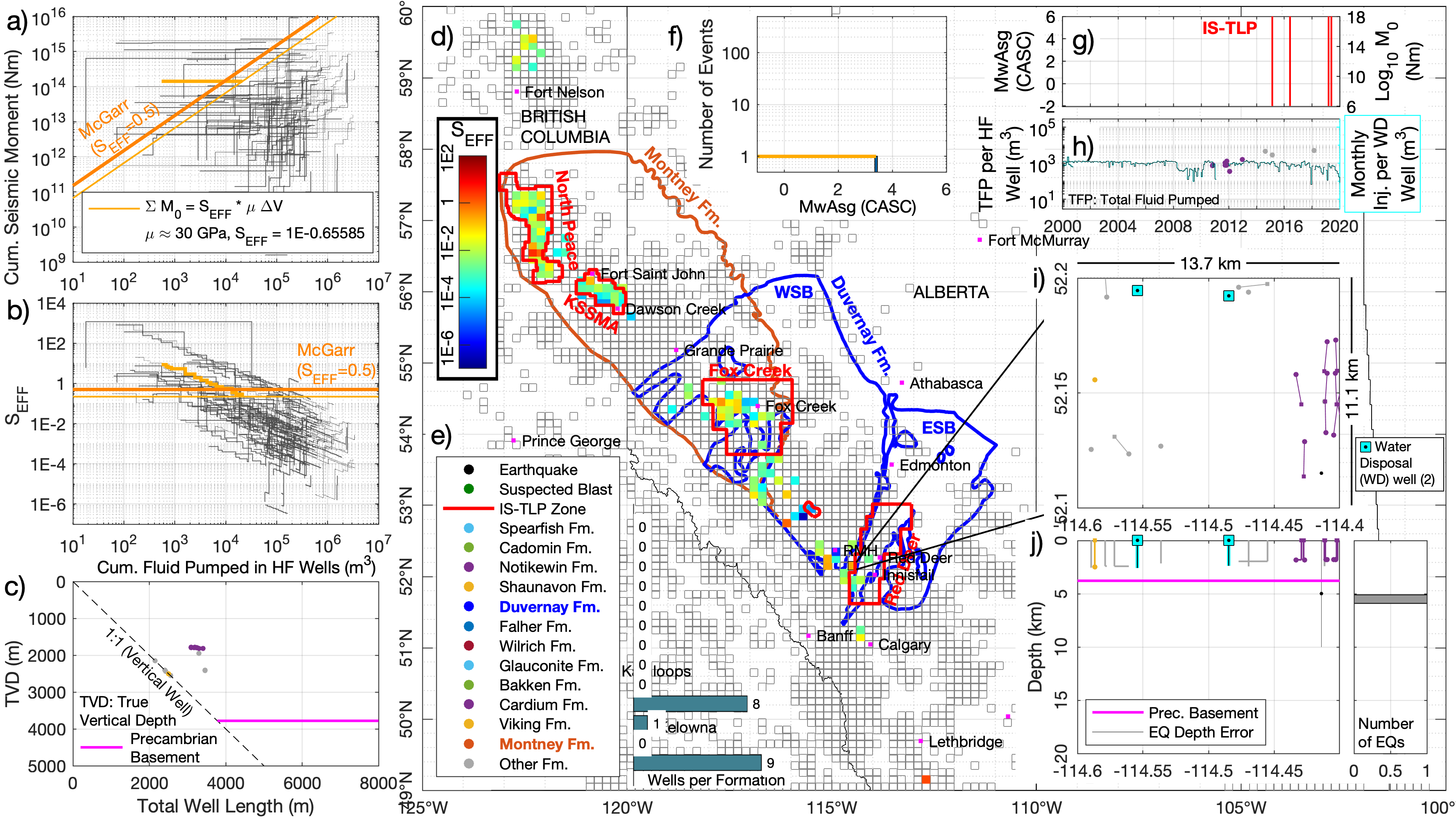

Supplement: Supplementary file 6 — Supplementary Information 6. [file 41598_2022_18505_MOESM6_ESM.zip › Figure S5 to S18 - 14 cases of runaway rupture/Figure S16 - AllFracs_CumTFP_CumM0_VariableSeff_Map_Cell_115.png]

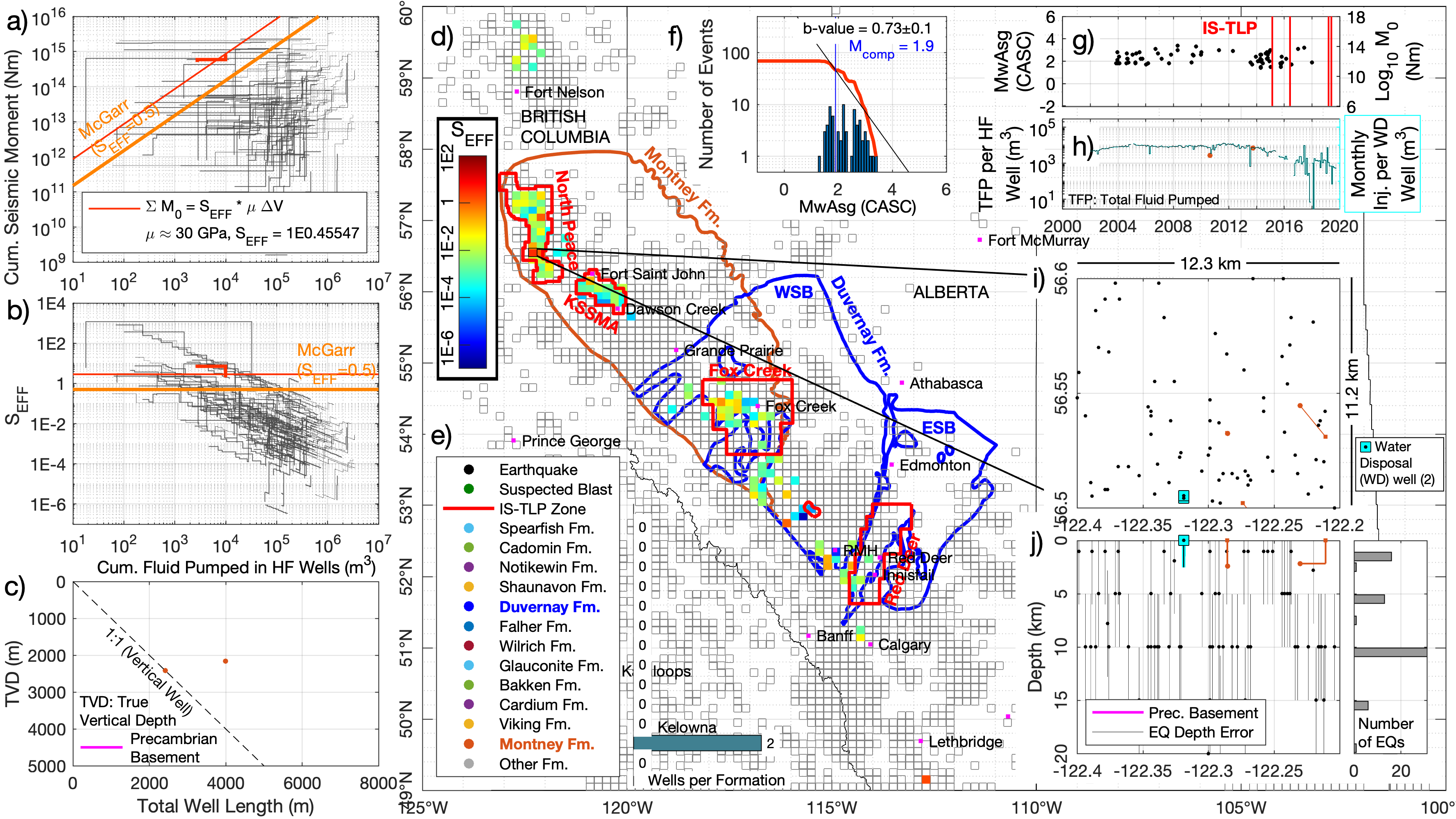

Supplement: Supplementary file 6 — Supplementary Information 6. [file 41598_2022_18505_MOESM6_ESM.zip › Figure S5 to S18 - 14 cases of runaway rupture/Figure S7 - AllFracs_CumTFP_CumM0_VariableSeff_Map_Cell_38.png]

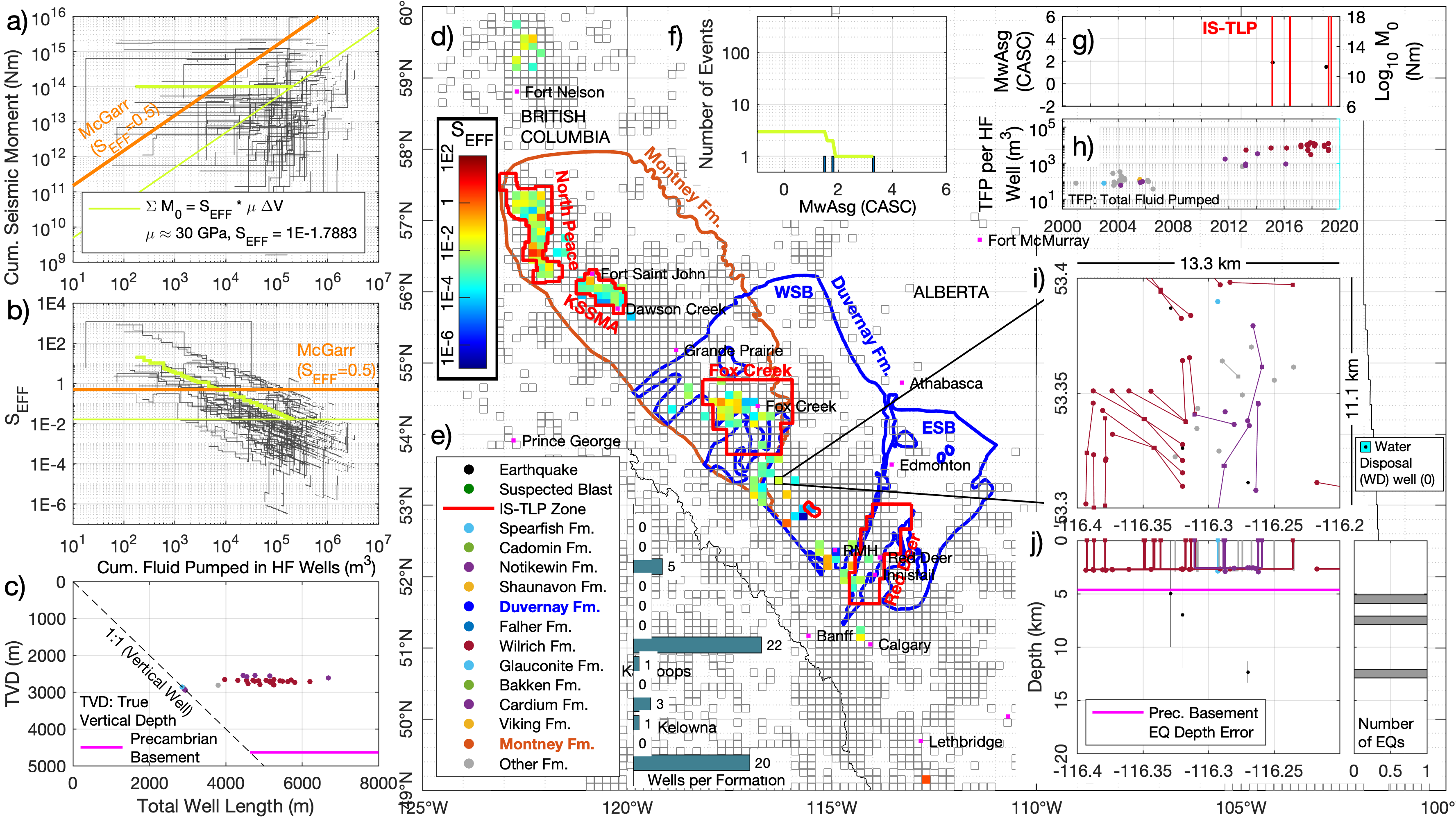

Supplement: Supplementary file 6 — Supplementary Information 6. [file 41598_2022_18505_MOESM6_ESM.zip › Figure S5 to S18 - 14 cases of runaway rupture/Figure S10 - AllFracs_CumTFP_CumM0_VariableSeff_Map_Cell_95.png]

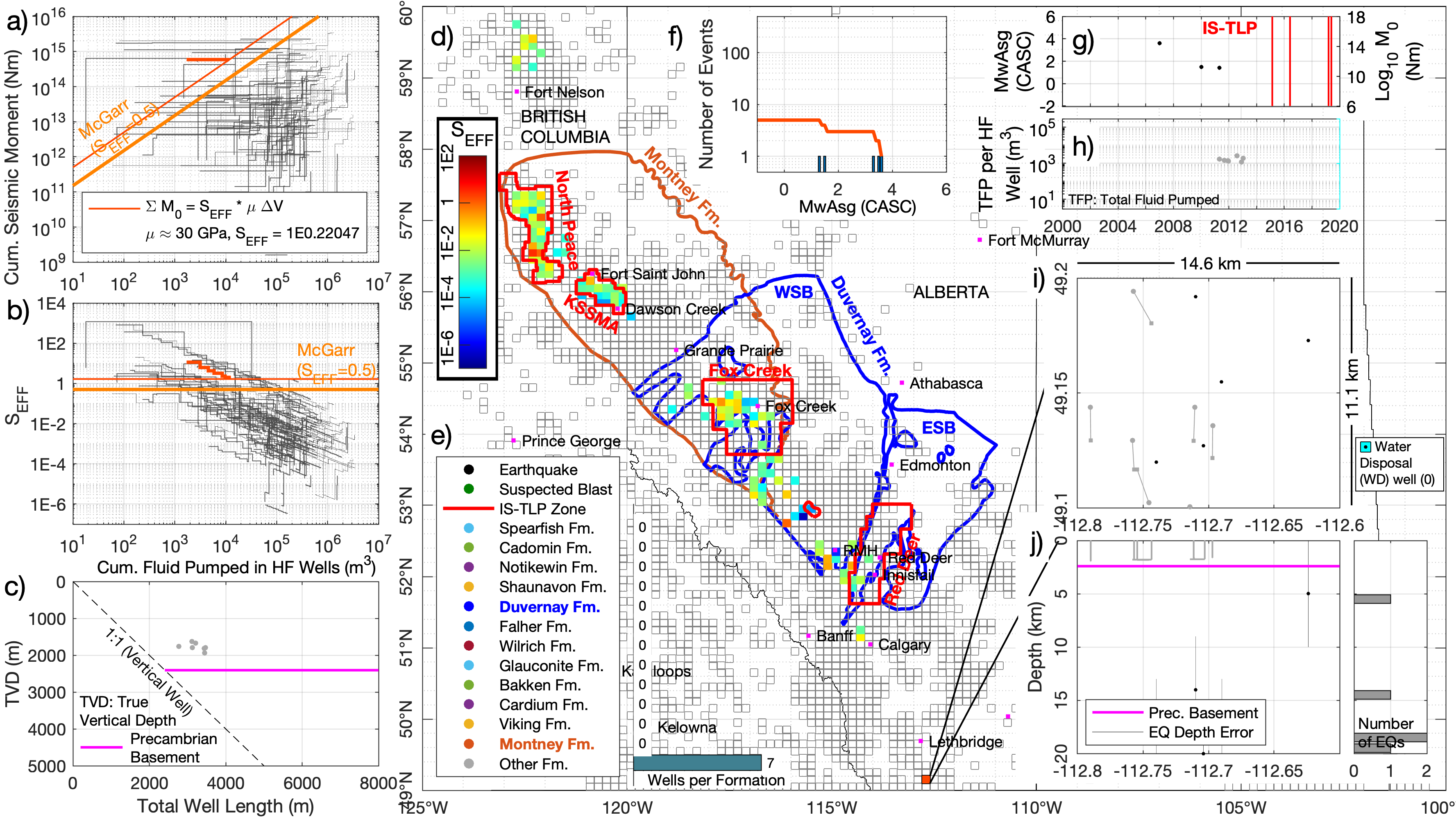

Supplement: Supplementary file 6 — Supplementary Information 6. [file 41598_2022_18505_MOESM6_ESM.zip › Figure S5 to S18 - 14 cases of runaway rupture/Figure S18 - AllFracs_CumTFP_CumM0_VariableSeff_Map_Cell_124.png]

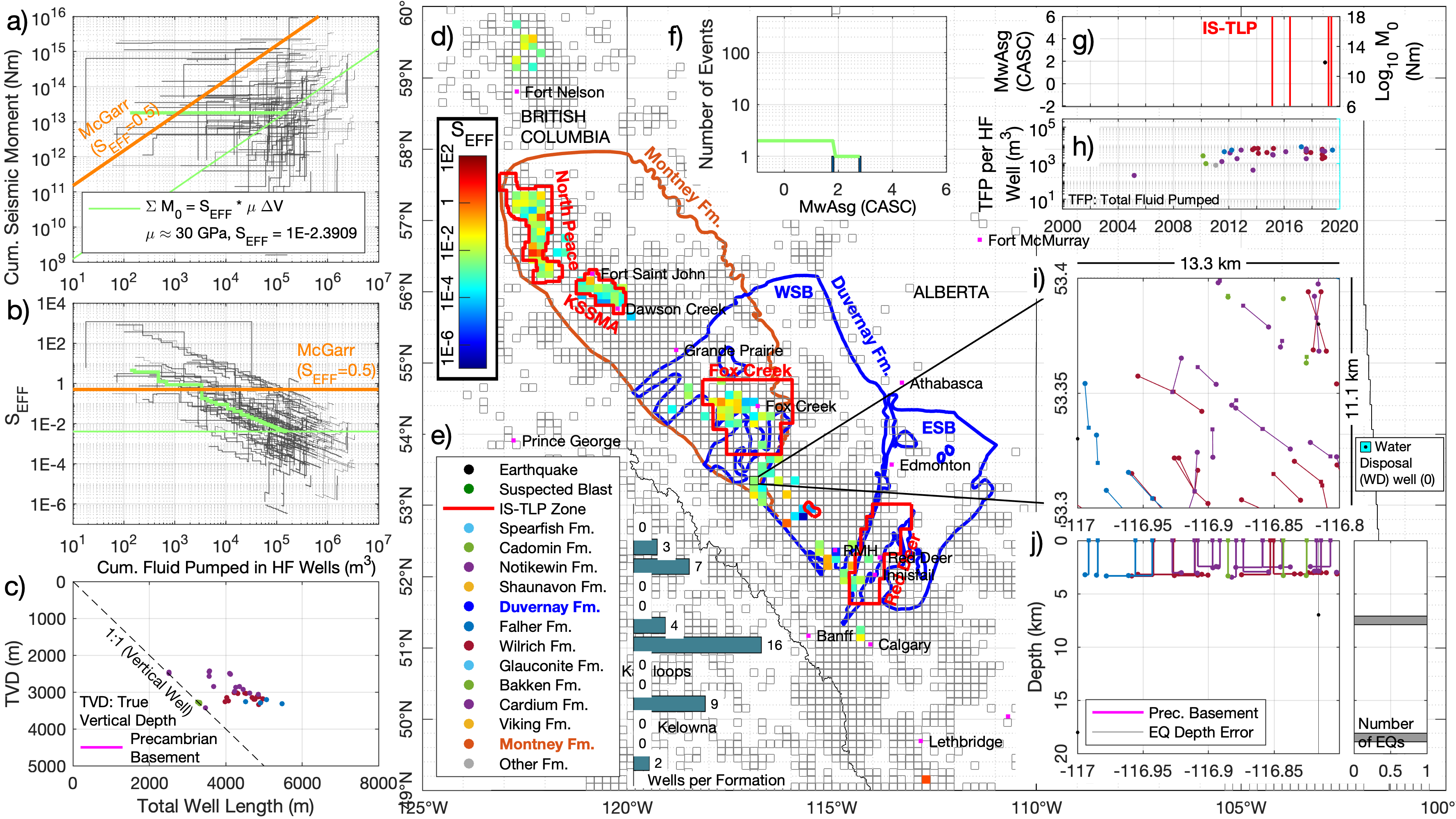

Supplement: Supplementary file 6 — Supplementary Information 6. [file 41598_2022_18505_MOESM6_ESM.zip › Figure S5 to S18 - 14 cases of runaway rupture/Figure S11 - AllFracs_CumTFP_CumM0_VariableSeff_Map_Cell_96.png]

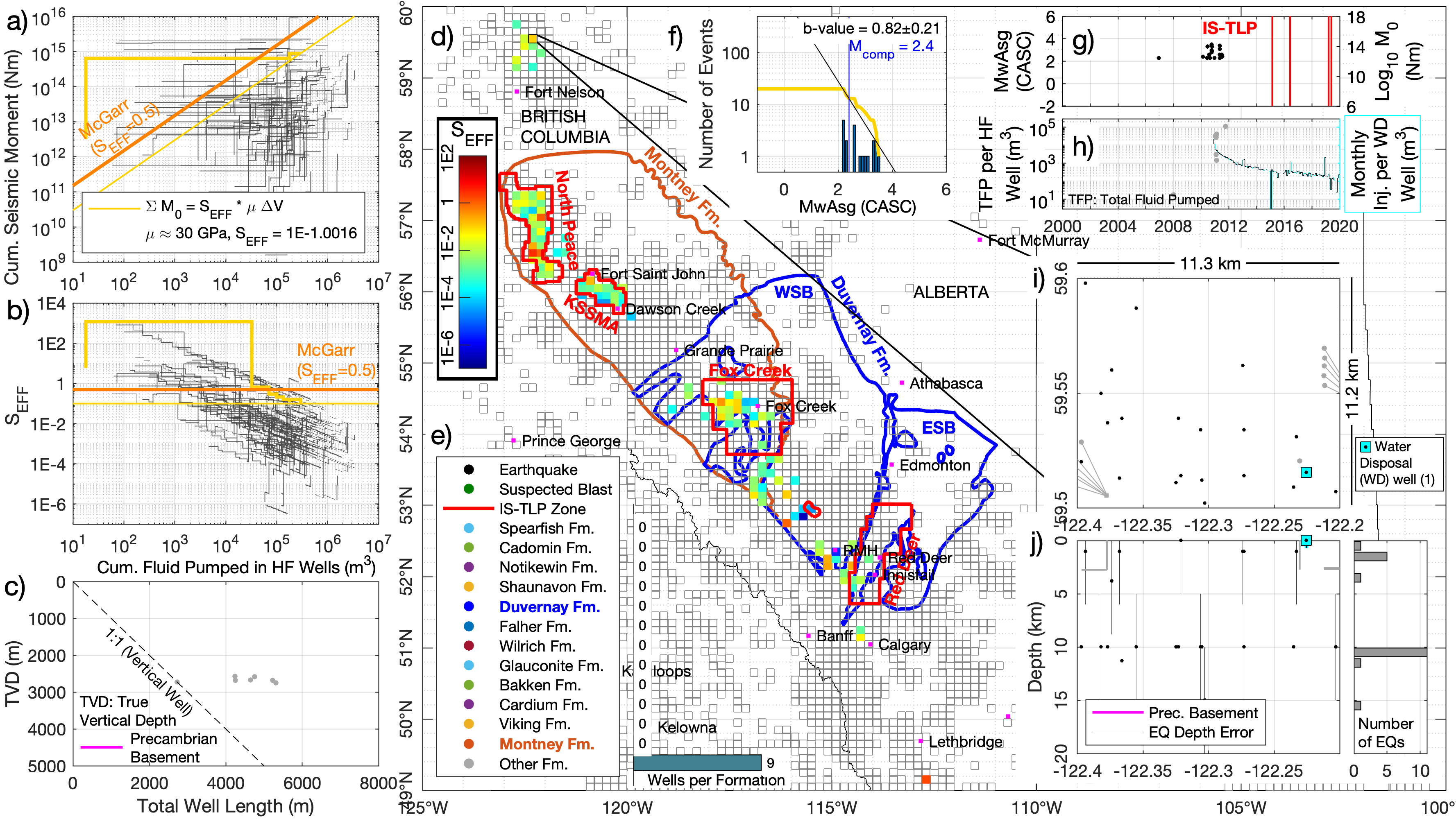

Supplement: Supplementary file 6 — Supplementary Information 6. [file 41598_2022_18505_MOESM6_ESM.zip › Figure S5 to S18 - 14 cases of runaway rupture/Figure S5 - AllFracs_CumTFP_CumM0_VariableSeff_Map_Cell_2.png]

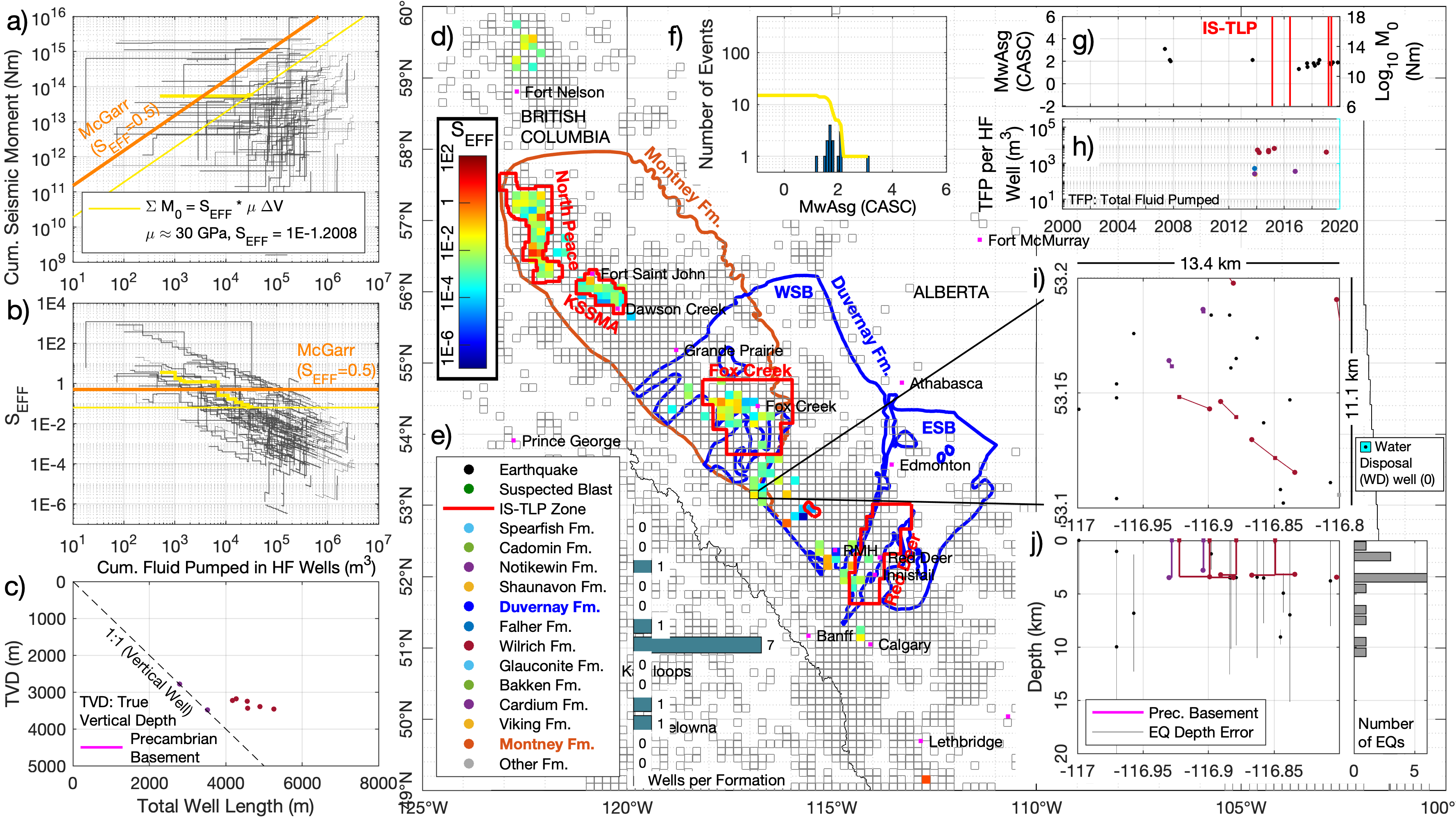

Supplement: Supplementary file 6 — Supplementary Information 6. [file 41598_2022_18505_MOESM6_ESM.zip › Figure S5 to S18 - 14 cases of runaway rupture/Figure S13 - AllFracs_CumTFP_CumM0_VariableSeff_Map_Cell_101.png]

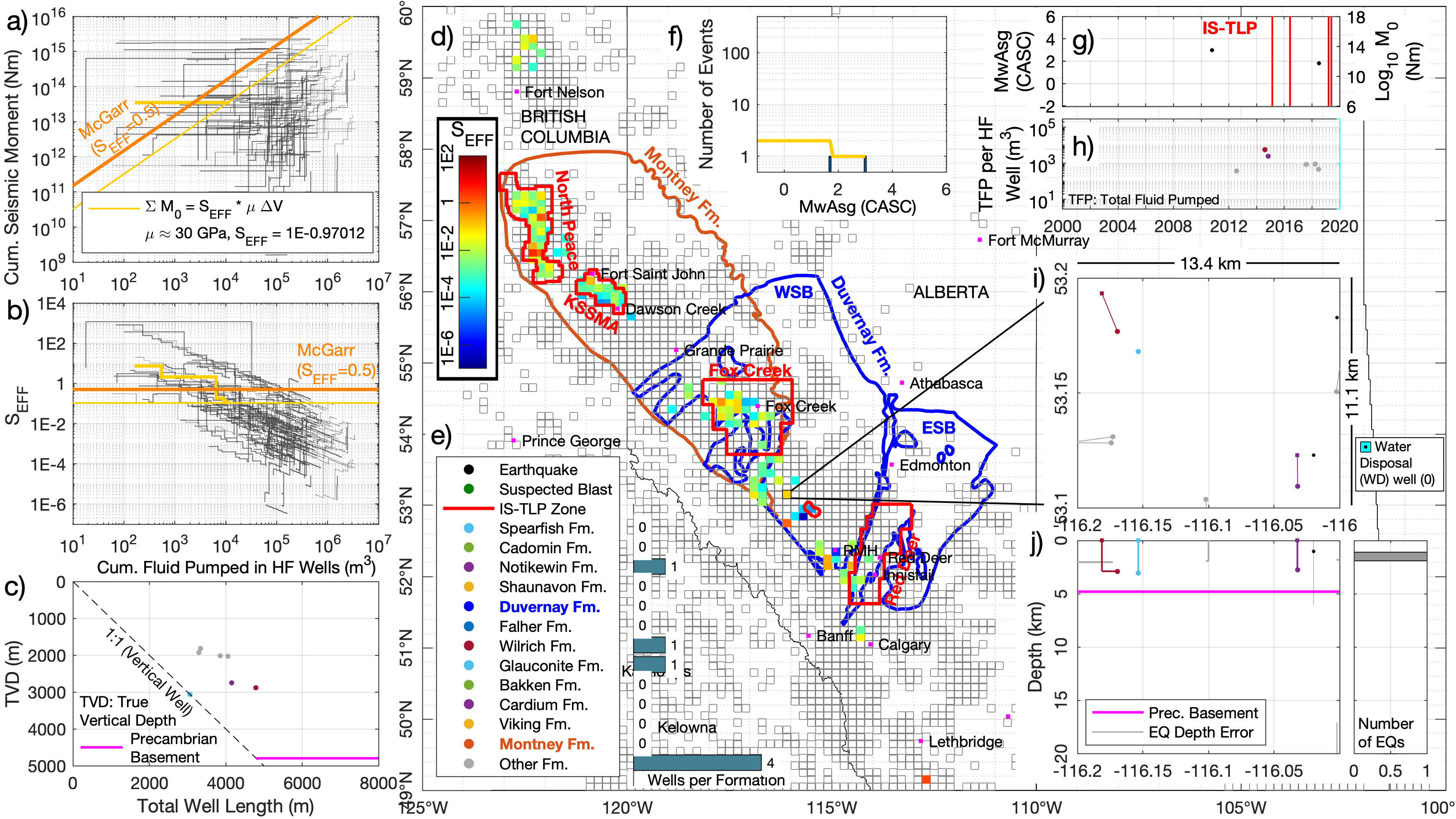

Supplement: Supplementary file 6 — Supplementary Information 6. [file 41598_2022_18505_MOESM6_ESM.zip › Figure S5 to S18 - 14 cases of runaway rupture/Figure S12 - AllFracs_CumTFP_CumM0_VariableSeff_Map_Cell_99.png]

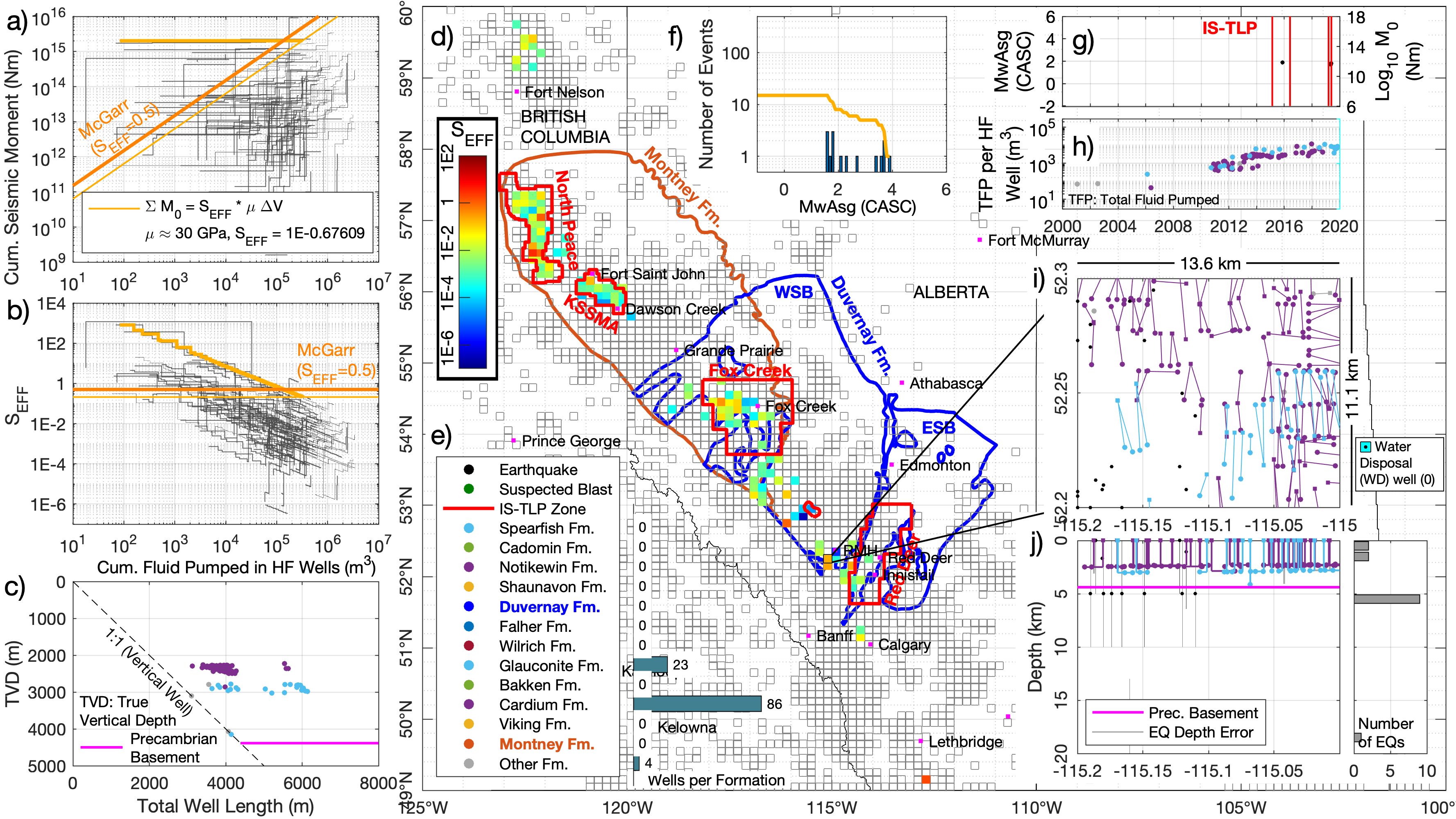

Supplement: Supplementary file 6 — Supplementary Information 6. [file 41598_2022_18505_MOESM6_ESM.zip › Figure S5 to S18 - 14 cases of runaway rupture/Figure S15 - AllFracs_CumTFP_CumM0_VariableSeff_Map_Cell_114.png]

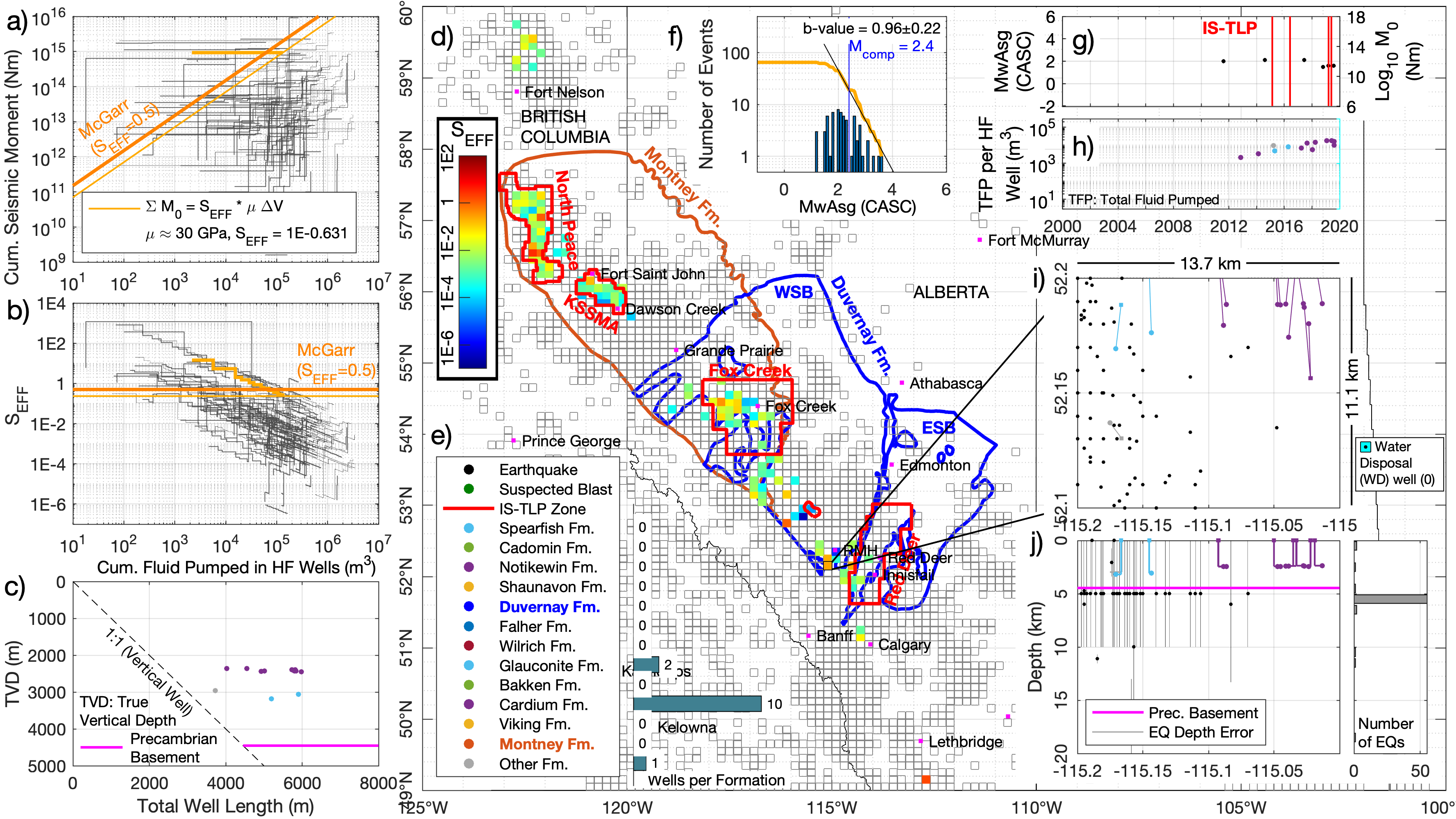

Supplement: Supplementary file 6 — Supplementary Information 6. [file 41598_2022_18505_MOESM6_ESM.zip › Figure S5 to S18 - 14 cases of runaway rupture/Figure S17 - AllFracs_CumTFP_CumM0_VariableSeff_Map_Cell_116.png]
